# Development of a two-stage limb ischemia model to better simulate human peripheral artery disease

**DOI:** 10.1038/s41598-020-60352-4

**Published:** 2020-02-26

**Authors:** Smriti M. Krishna, Safraz Mohamed Omer, Jiaze Li, Susan K. Morton, Roby J. Jose, Jonathan Golledge

**Affiliations:** 1 The Vascular Biology Unit, Queensland Research Centre for Peripheral Vascular Disease, School of Medicine and Dentistry, James Cook University, Townsville, Queensland 4811 Australia; 2 Department of Vascular and Endovascular Surgery, The Townsville Hospital, Townsville, Queensland 4811 Australia

**Keywords:** Experimental models of disease, Translational research

## Abstract

Peripheral arterial disease (PAD) develops due to the narrowing or blockage of arteries supplying blood to the lower limbs. Surgical and endovascular interventions are the main treatments for advanced PAD but alternative and adjunctive medical therapies are needed. Currently the main preclinical experimental model employed in PAD research is based on induction of acute hind limb ischemia (HLI) by a 1-stage procedure. Since there are concerns regarding the ability to translate findings from this animal model to patients, we aimed to develop a novel clinically relevant animal model of PAD. HLI was induced in male Apolipoprotein E (*ApoE*^*−/−*^) deficient mice by a 2-stage procedure of initial gradual femoral artery occlusion by ameroid constrictors for 14 days and subsequent excision of the femoral artery. This 2-stage HLI model was compared to the classical 1-stage HLI model and sham controls. Ischemia severity was assessed using Laser Doppler Perfusion Imaging (LDPI). Ambulatory ability was assessed using an open field test, a treadmill test and using established scoring scales. Molecular markers of angiogenesis and shear stress were assessed within gastrocnemius muscle tissue samples using quantitative polymerase chain reaction. HLI was more severe in mice receiving the 2-stage compared to the 1-stage ischemia induction procedure as assessed by LDPI (*p* = *0.014*), and reflected in a higher ischemic score (*p* = *0.004*) and lower average distance travelled on a treadmill test (*p* = *0.045*). Mice undergoing the 2-stage HLI also had lower expression of angiogenesis markers (vascular endothelial growth factor, *p* = *0.004*; vascular endothelial growth factor- receptor 2, *p* = *0.008*) and shear stress response mechano-transducer transient receptor potential vanilloid 4 (*p* = *0.041*) within gastrocnemius muscle samples, compared to animals having the 1-stage HLI procedure. Mice subjected to the 2-stage HLI receiving an exercise program showed significantly greater improvement in their ambulatory ability on a treadmill test than a sedentary control group. This study describes a novel model of HLI which leads to more severe and sustained ischemia than the conventionally used model. Exercise therapy, which has established efficacy in PAD patients, was also effective in this new model. This new model maybe useful in the evaluation of potential novel PAD therapies.

## Introduction

Peripheral arterial disease (PAD) leads to impaired lower limb blood supply usually as a result of atherosclerosis and associated thrombosis^1^. In 2010 an estimated 200 million people worldwide were living with PAD, an increase of ~30% since 2000 which has been referred to as a PAD pandemic^2,3^. The burden of PAD is increasing worldwide and particularly within low and middle-income countries^4^. PAD is associated with significant morbidity including intense leg pain during walking (intermittent claudication, IC), impaired walking ability, poor health-related quality of life and risk of serious complications such as major leg amputation and death^1^. Medical management of PAD is mainly focused on reducing the risk of cardiovascular events (through statins, blood pressure-lowering and anti-platelet medication prescription), rather than treating leg symptoms^2^. The only drug therapy available for IC is cilostazol^5^. The benefit of this drug have been deemed too small in many countries for it to be publically funded. In high-income countries the most widely used treatments for advanced PAD are surgical approaches to restoring the arterial blood supply to the legs using temporary balloons, permanent stents or open surgical bypasses. Stents and other peripheral revascularisation procedures have a risk of serious complications (such as major amputation, renal failure and death) and poor long-term durability^5–8^. There is great interest in developing novel medical therapies for the leg symptoms of PAD. Recent efforts have focused on stimulating development of new blood vessels within the leg through angiogenesis or by encouraging the remodelling of existing small vessels into improved collateral channels (arteriogenesis). Promising results for novel treatments, such as viral vectors carrying angiogenesis promoting agents and stem cells, in pre-clinical models of PAD have not been consistently replicated in large clinical trials^9–12^.

In patients that have PAD, atherosclerosis-associated arterial narrowing develops gradually over many years allowing the legs to adjust to the gradual decrease in blood flow through compensatory mechanisms within the blood vessels and muscle fibres^13^. In contrast, the most commonly used animal model for initial testing of novel therapies for PAD is a model of acute blood supply interruption through ligation or excision of the femoral artery (referred to here as the 1-stage hind limb ischemia (HLI) model)^14,15^. Previous studies report that the ligation and excision of the femoral artery in the 1-stage model leads to increased fluid shear stress within the limb collateral arteries resulting in altered gene expression patterns through shear stress responsive elements which promote arterio- and angio-genesis^16–18^. Hind limb blood supply in this 1-stage model therefore usually naturally recovers over a period of approximately 4 weeks^15^. This model does not therefore simulate the clinical presentation of PAD. Patients typically present with a history of acute exacerbation of chronic symptoms of leg pain on walking and have ongoing ischemic symptoms. The 1-stage HLI model may therefore not be an ideal model to study therapeutic angiogenesis and arteriogenesis^14,19^. Another approach to inducing HLI is the placement of an ameroid constrictor around the femoral artery to induce gradual occlusion^16,20,21^. Previous studies suggest that this approach leads to mild ischemia^20^ and that blood flow recovery occurs within 2–3 weeks^16,21^. Furthermore, previous pre-clinical PAD research has mainly focused on assessing hind limb blood supply with limited assessment of ambulatory ability^14,15^. On the other hand, the assessment of novel treatments in PAD patients usually involves measures of walking ability using treadmill or corridor walking tests^22,23^. There is therefore a need for an increased focus on functional tests of the limb within clinically-relevant rodent models.

We hypothesised that the limb ischemia produced by the current 1-stage HLI model would be more severe and sustained if the model was modified to include an initial more slowly progressive arterial narrowing over 14 days prior to the induction of acute ischemia (i.e. a 2-stage model). Our overall aim was to develop a more clinically relevant rodent model that could incorporates stable on-going limb ischemia in order to test therapeutic interventions.

## Materials and Methods

### Mice

Male Apolipoprotein E deficient (*ApoE*^*−/−*^) mice (n = 118, obtained from Animal Resources Centre, Western Australia) were used for the experiments. Mice were housed in a temperature-controlled room (21 ± 1 °C) with an automatic 12:12-h light/dark cycle (07:00 to 19:00 hours). Mice were singly housed in a clear individually-ventilated, temperature and humidity-controlled cage system (Aero IVC Green Line; Tecniplast) with enrichment. All experiments were performed during the light phase (10:00–18:00 hours) and mice were fed with standard rodent chow and water *ad libitum* during the course of these experiments. Approval for the animal studies was obtained from the institutional ethics committee (Animal Ethics Committee, James Cook University) and experimental work performed in accordance with the institutional and ethical guidelines of James Cook University, Australia, and conforming to the Guide for the Care and Use of Laboratory Animals (National Institutes of Health, USA).

### HLI models

**Figure 1:**
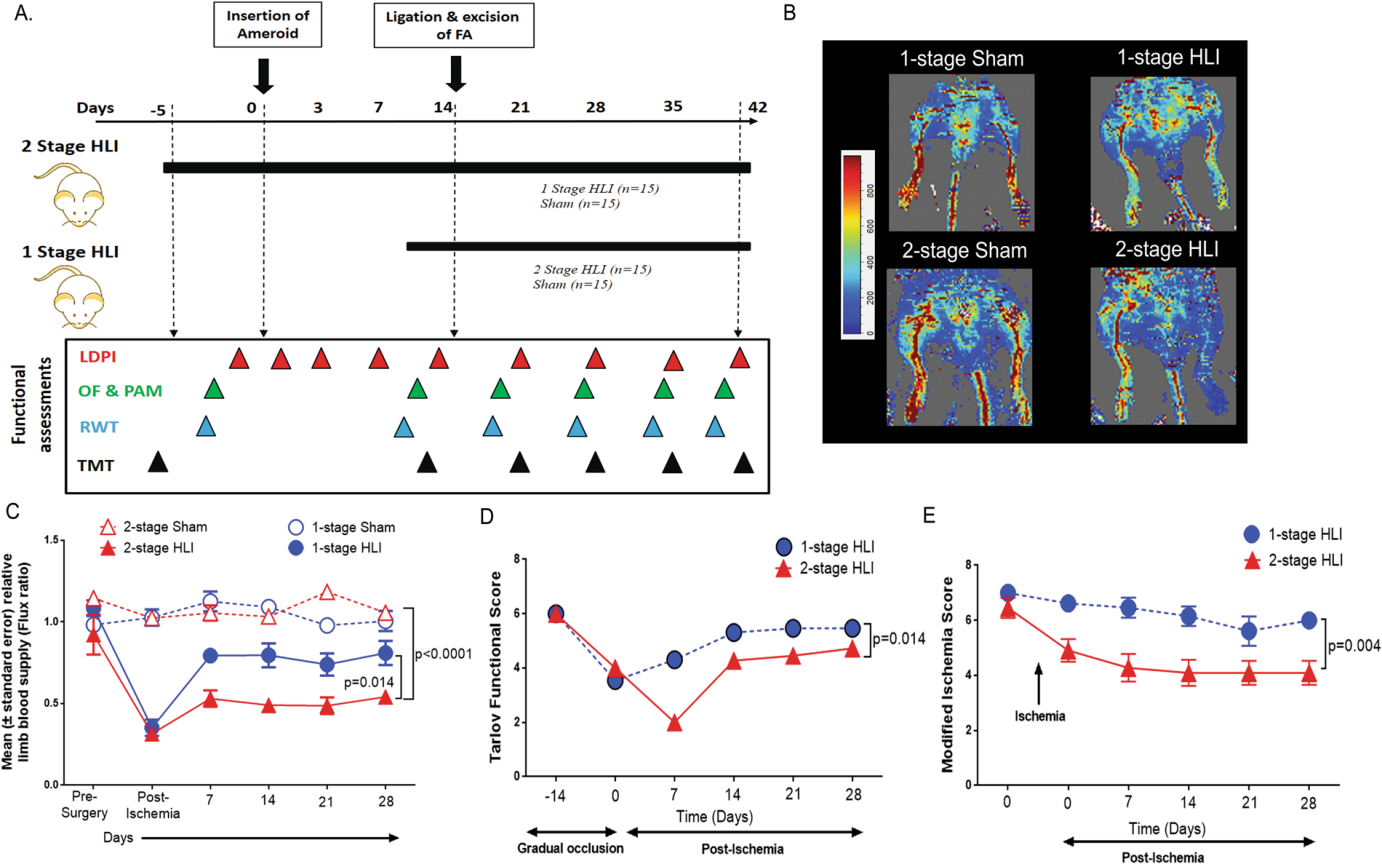
A surgically induced 2-stage HLI model showed reduced perfusion and functional score after ischemia induction compared to controls. (**A**) Schematic representation of the experiment and surgery showing the time points of various assessments; mainly Laser Doppler Perfusion Imaging (LDPI), Open field (OF), Pain application measurement (PAM), Running wheel test (RWT) and Treadmill test (TMT). (**B**) Representative LDPI images obtained 28 days after ischemia induction in mice undergoing the 1-stage and 2-stage Hind limb ischemia (HLI) models with their respective controls. (**C**) Quantitative image analysis showing the ratio of the ischemic to contralateral limb perfusion following different HLI models. The 2-stage HLI model displayed delayed restoration of perfusion compared to the 1-stage HLI model. Data shown as mean ± SEM and analysed by repeated measures 2-way ANOVA. (**D**) Graph showing Tarlov functional scores. The movement of animals were monitored and functional scores of the various groups were assessed according to the scoring criteria detailed in the materials and methods section. The 2-stage HLI model showed reduced function throughout the experimental period compared to the 1-stage HLI model. Data shown as mean ± SEM and analysed by repeated measures 2-way ANOVA, and p value significant at ≤0.05. (**E**) Graph showing modified ischemia scores. The animals were monitored and scored for signs of ischemia according to previously published criteria detailed in the materials and methods. The 2-stage HLI model showed a higher level of ischemia compared to the 1-stage HLI depicted by a lower scoring throughout the study period. Data shown as median ±SEM and analysed by repeated measures 2-way ANOVA, and p value significance set at ≤*0.05*.

The first phase of the study utilised two HLI models: the most commonly used unilateral acute HLI model (1-stage HLI)^16,24,25^ and the newly developed 2-stage HLI model. Male *ApoE*^*−/−*^ mice aged 20 months were randomly divided into 4 groups as follows: group 1 = 1-stage HLI model (n = 25), group 2 = 1-stage sham (n = 19), group 3 = 2-stage HLI model (n = 25) and group 4 = 2-stage sham (n = 19). Body weight and primary outcome measures were recorded at regular intervals as illustrated in Fig. 1A. All functional assessments were performed in a subset of mice randomly selected from each experimental group (n = 5–7). The creation of the 1-stage HLI model involved exposure of the left femoral artery through a vertical 0.5–1 cm skin incision under a stereotactic microscope (Leica). The femoral artery and its side branches were then ligated with 6–0 silk sutures (Ethicon) immediately distal to the inguinal ligament and proximal to the popliteal bifurcation before being excised (Supplementary Fig. S1A,B). Femoral nerves were carefully preserved. The wound was irrigated with sterile saline and then the overlying skin was closed using 4–0 vicryl sutures (Ethicon). Post-operative pain was reduced using Lignocaine (Troy Laboratories). A similar surgery without ligation or excision of the femoral artery was performed on the 1-stage sham controls.

The 2-stage HLI model was performed using a 2-stage surgical procedure. The left femoral artery was exposed as described above and 2 custom made miniature ameroid constrictors of 0.25 mm internal diameter (Research Instruments SW) were positioned on the artery. One was placed on the femoral artery immediately distal to the inguinal ligament and one was positioned proximal to the sapheno-popliteal bifurcation (Supplementary Fig. S1C). After 14 days, a new incision was made and the femoral artery ligated and excised, as described for the 1-stage HLI model. A similar two-stage surgery was performed without placement of ameroids, nor ligation and excision of the femoral artery for the 2-stage sham controls.

### Assessment of the effect of exercise training in the 2-stage HLI model

During the second phase of the study, the effect of an exercise program on male *ApoE*^*−/−*^ mice aged 6 months undergoing the 2-stage HLI model (male *ApoE*^*−/−*^ mice, n = 30) was tested. Mice subjected to 2-stage HLI were randomly allocated to an exercise training or control group (n = 15 per group). Mice in the exercise group received 180 to 200 m (between 30–45 mins of running wheel access) of exercise each day on a running wheel (8 Station Home Cage Running Wheel System, Columbus Instruments) for 4 weeks. Each mouse was placed on a running wheel within a small chamber, which prevented the mouse from escaping the running wheel during the exercise period.

### Laser doppler perfusion imaging

Blood flow was measured noninvasively by the laser Doppler perfusion imaging (LDPI, *moorLDI2*, Moor Instruments) system which has been validated extensively and is considered a reference standard for perfusion assessment^26,27^. Briefly, the assessment of limb perfusion was by acquiring flow images of the ischemic and contralateral limb. All of the mice were evaluated under the same experimental conditions and were scanned from the lower abdomen to the end of the toes. During imaging, ambient light and temperature (maintained at 37 °C core temperature) were carefully controlled to avoid background variations. Hair was removed by epilating cream on the day prior to LDPI measurements, mice were placed in dorsal decubitus and anaesthetised using isoflurane (2.0% induction dose and 1.8% maintenance dose plus oxygen; 1 L/min, VET Equip). The LDPI system was mounted on a movable track that was fixed 26 cm above the mice limbs while the animals were restrained on a warming table with a black surface. Image analysis software (*Laser Doppler Perfusion Measure, V3.08*, Moor Instruments) was used to calculate the limb mean flux units, which represents a quantitative analysis of tissue perfusion on a scale of 0 to 1000 perfusion units (PU). Identical regions of both ischemic and contralateral non-ischemic limbs were assessed for quantification of perfusion. LDPI images were analysed by two independent observers blinded to the experimental group and averaged. Limb perfusion was expressed as the ratio of the flux value of the ischemic (left) limb relative to the value of the contralateral non-ischemic (right) limb in the same mouse and presented as average perfusion units (PU). Previous reports of the 1-stage HLI model suggested that the HLI typically resolved over the course of 28 days, with hind limb blood supply reaching a plateau between 21 and 28 days after surgery^28^. The LDPI measurements were therefore performed at the following time points for 1-stage HLI model: day 0 prior to surgery, immediately after surgery, days 3, 7, 14, 21 and 28 after surgery. For the 2-stage HLI model, the LDPI measurements were performed at the following time points: day 0 prior to the first operation (ameroid placement), immediately after the first operation, days 3, 7, and 14 after the first operation, immediately after the second operation (femoral artery excision), and 7, 14, 21 and 28 days after the second operation (i.e. 14, 21, 28, 35 and 42 days after the first operation). A schematic illustration of the experimental design is shown in Fig. 1A. Body mass was also measured on the same day as the LDPI measurements.

### Secondary outcomes

In clinical practice PAD patients are treated to improve pain free walking capacity and resolve rest pain and tissue loss (critical limb ischemia, CLI). In clinical trials, these are usually investigated by walking tests and assessment of pain. Similar to clinical trials, in the current study ambulatory ability was assessed with a treadmill test, voluntary physical activity examined through an open field test and foot pain estimated through a mechanical allodynia test. All outcomes were assessed by an assessor blinded to mice group.

#### Assessment of limb function and ischemia

Semi-quantitative assessments of limb function and ischemia were performed at the same time points as the blood flow measurements (Fig. 1A; Supplementary Tables S1, S2). Limb function was assessed using the clinical use score (Tarlov scale) as: 0 = no movement; 1 = barely perceptible movement, no weight bearing; 2 = frequent and vigorous movement, no weight bearing; 3 = supports weight, may take 1 or 2 steps; 4 = walks with only mild deficit; 5 = normal but slow walking and 6 = full and fast walking^29,30^. Limb ischemia was scored using the ischemia scoring scale as previously reported: 0 = auto-amputation of leg; 1 = leg necrosis; 2 = foot necrosis; 3 = two or more toe discoloration; 4 = one toe discoloration; 5 = two or more nail discolorations; 6 = one nail discoloration and 7 = no necrosis^30^. All scoring was performed by two independent observers and found to be identical.

#### Treadmill test

Mice were run on a six lane Excer3/6 Treadmill (Columbus Instruments) without incline. Mice were acclimatised to the treadmill by ambulating on it at 5 m/min for 5 min once daily on three consecutive days prior to any testing. Before each treadmill test, mice were fasted for 1 h. The speed of the treadmill was controlled using the software and calibrated using an inbuilt speedometer mounted on the treadmill platform. A treadmill test involved an initial warm up at 5 m/min for 5 min followed by a progressive speed increase from 5 to 10 m/min, accelerated at 1 m/min. Following this the treadmill speed remained at 10 m/min for up to a total running time of 20 min. During the test a stimulus grid of 3 Hz was kept on until mouse exhaustion as previously reported^31^. Exhaustion of the mouse was defined if the mouse returned to the stimulus grid 10 times despite a 3 Hz electrical stimulus to encourage walking on the belt. The treadmill software recorded the total distance walked by a mouse until exhaustion. A blinded observer supervised the experiment to assess outcomes. The treadmill belt and lanes were cleaned with water and 70% alcohol and dried with paper towel after each test to remove any body scent. Treadmill testing was carried out before ameroid placement, 10 days after ameroid placement, and 1 and 3 weeks after completion of the 2-stage HLI (Fig. 1A). For the 1-stage HLI model, treadmill testing was performed before ligation and excision of the femoral artery and 1 and 3 weeks after ischemia induction.

#### Voluntary physical activity test

The open field test is a common measure of voluntary physical activity in rodents suggested to be similar to a 6-min walk test used in humans^32^. To ensure consistency prior to the test, mice were brought to the testing room in their home cages at least 1 hr prior to the start of behavioural testing. The mice were fasted during the acclimatisation period and given free access to water under normal lighting. The open field box was made of opaque plastic (40 × 40 × 40 cm), divided into an outer field (periphery) and a central field (20 × 20 cm) which was primarily used for analysis. Mice were individually placed in the centre of the arena and movements of the mice were recorded using a video camera (Logitech) supported with acquisition software (*Capture Star Ver. 1*; CleverSys Inc) and analysed by the TopScan Lite software (*High throughput version 2.0*; CleverSys Inc). The test protocol used was identical for each mouse assessed. After each test the open field box was cleaned with water and 70% alcohol and dried with a paper towel to remove the body scent, which could be a cue to movement of the mice. Room lighting, temperature, and noise levels were kept consistent for all tests. The mouse movements were recorded for 20 min, to mimic the short timed nature of the 6-min walk test. Rest time was recorded as motion measure score <0.05 in the software and average speed was calculated only for motion measure score >0.05. Total distance travelled (m), frequency of movement, time spent in the arena (s) and average velocity in the arena (mm/s) were measured.

#### Mechanical allodynia test

The paw pressure transducer and the pressure application measurement device (PAM; Ugo Basile) is a non-invasive tool for measuring mechanical allodynia threshold and hypersensitivity in rodents. The PAM device allows an accurate measurement of primary mechanical hypersensitivity in rodents^33,34^. A gradually increasing squeeze force is applied across the joint at a rate of approximately 300 gms/sec until a behavioural response (paw withdrawal, freezing of whisker movement, wriggling or vocalization) is observed with a cut-off of 5 sec. The peak gram force (gf) applied immediately prior to limb withdrawal was recorded by the base unit, and this value was designated the limb withdrawal threshold (LWT). LWT was measured twice in both the ipsilateral and contralateral limbs by two independent observers. The measurements were averaged and presented as a ratio of operated left limb to the un-operated right limb.

### Blood tests

Blood was collected by cardiac puncture at the completion of the experiments. Platelet poor plasma was separated as described previously^35,36^. The plasma concentrations of interleukin (IL)-6, interferon (IFN)-γ, monocyte chemoattractant protein-1 (MCP-1) and tumour necrosis factor (TNF)-α were determined using a cytometric bead array kit (CBA, BD Biosciences). The inflammatory markers were assessed in samples (n = 10/group) selected from each group using a random number generator. Briefly, 50 μl of mixed capture beads and 50 μl of serially diluted standard or plasma sample and 50 μl of phycoerythrin (PE) detection reagent, were incubated in the dark for 2 h in sample assay tubes. Samples were then washed twice with 1 ml of the wash buffer, resuspended, and acquired on the Cyan ADP flow cytometer (Beckman Coulter). Results were analysed and quantified by FCAP Array™ software (*v3*, BD Biosciences). We previously reported this method to have good reproducibility with an inter-assay coefficient of variation of 6–9% (n = 8–10)^36^. Total nitrate was measured in plasma samples by a nitrate/nitrite colorimetric assay kit following the manufacturer’s protocol (inter-assay coefficient of variation 2.7%; Cayman Chemicals) as reported previously^37^. Briefly, nitrate was converted to nitrite using nitrate reductase. Subsequently, addition of the Griess reagents converted nitrite into a deep purple azo compound and the absorbance was measured at 540 nm using an Omega plate reader.

### Histological assessments

Low capillary density has been reported in the gastrocnemius muscle of PAD patients and animal HLI models and associated with functional impairment^38,39^. Hence at the end of experiments gastrocnemius muscle samples were collected from the mice and stored in optimal cutting compound (OCT, ProSciTech) which was progressively frozen in isopentane (Sigma) suspended in liquid nitrogen. Sections (5 µm-thick) were obtained on Poly L-Lysine coated slides (ProSciTech) from each sample with muscle fibres oriented in the transverse direction. All histological assessments were performed on sections that were examined in a blinded fashion at 40x magnification.

#### Muscle fibre structure

Fixed cryostat sections were stained with hematoxylin and eosin (H&E, ProSciTech), examined at magnifications of 100x or 400x to assess the integrity of the tissues. Degenerating muscle fibres were identified in the H&E stained sections by morphological assessment. Assessors looked for the presence of mature skeletal muscle fibres (small peripheral nuclei) versus immature skeletal muscle myoblasts (large lobulated central nuclei)^40^. Muscle fibre number and size were examined in 5 separate fields in 4 distinct areas in each specimen.

#### Muscle fibrosis

The extent of skeletal muscle fibrosis was assessed by staining the cryostat sections (5µm-thick) with picrosirius (ProSciTech). Briefly, tissue sections were stained and examined under 40x power light microscope and skeletal muscle fibrosis was analysed using the image analysis software (Zeiss Axio Imager *Z1*). Quantification of fibrosis was expressed as the percentage of fibrotic tissue present within the section (1 mm^2^ tissue area) using a previously published protocol^40^.

#### Immunohistochemistry and morphometric analysis of capillary and arteriolar density

These were performed as previously reported^41,42^. The gastrocnemius muscles from ischaemic and non-ischaemic hind-limbs were collected and embedded in OCT compound (ProSciTech), frozen, and cut into 5 µm-thick sections. The slides were fixed at −20 °C in 95% ethanol for 1 hr. Slides were washed three times in cold PBS with 1% horse serum (5 min/wash) and blocked overnight with 5% horse serum in PBS at 4 °C. Immunohistochemistry was performed using primary antibodies against CD31 (1:100 dilution; Abcam) and smooth muscle α-actin (α-SMA, 1:200 dilution; Abcam). Bound primary antibodies were detected by using appropriate secondary antibodies (biotinylated anti-goat IgG and biotinylated anti-Rat IgG, all at 1:100 dilutions, Vector Labs) using avidin-biotin-peroxidase (Vector Labs) as described previously^43^. Pictures from four random areas of each section and three sections per mouse were taken by using a digital camera (Nikon Eclipse *Sci* epifluorescence microscope, Nikon Corporation) at 40× magnification. Capillary density were quantified by measuring the percentage of CD31 and α-SMA staining out of the total area as previously described.

### Western blotting

Gastrocnemius muscles were mainly harvested at the end of studies (i.e. 4 weeks after full ischemia induction). Gastrocnemius muscles were also harvested from a subset of mice (n = 15) subjected to the 2-stage HLI (n = 5/time-point) prior to and days 3 and 7 after ameroid placement. Samples were frozen in liquid nitrogen, and stored in OCT compound (ProSciTech) at −80 °C. Tissues were pulverised in RIPA buffer (150 mM sodium chloride, 1.0% NP-40 or Triton X-100, 0.5% sodium deoxycholate, 0.1% sodium dodecyl sulfate, 50 mM Tris, pH 8.0) with protease inhibitors (Roche Diagnostics, Australia) and PhoSTOP (Roche Diagnostics, Australia) to extract proteins and quantitated using the Bradford protein assay kit (BioRad, USA). Samples (25 μg of protein/lane) were loaded onto a 10% SDS-polyacrylamide electrophoresis gel. After electrophoresis (110 V, 90 min), the separated proteins were transferred (15 mA, 60 min) to a polyvinylidene difluoride membrane (BioRad, USA). Non-specific sites were blocked with 5% non-fat dry milk for 60 min, and the blots were then incubated with following antibodies: anti-vascular endothelial growth factor (anti-VEGF, 1:1000; Cells Signalling, USA), anti-VEGF-Receptor 1 (anti-VEGF-R1, 1:1000; Cell Signalling, USA), anti-VEGF-R2 (1:1000; Sigma-Aldrich, USA), anti-phospho-endothelial nitric oxide synthase (anti-p-eNOS, 1:1000; Cell Signalling, USA), anti-eNOS (1:1000; Cell Signalling, USA), anti-hypoxia-inducible factor 1-alpha (anti-HIF-α, 1:1000; Cell Signalling, USA), anti-transient receptor potential cation channel subfamily V member-4 (anti-TRPV4, 1:1000; Sigma-Aldrich, USA) and anti-kruppel like factor-4 (anti-KLF4, 1:1000; R&D), overnight at 4 °C. Anti-rabbit HRP conjugated IgG (1:1000; DakoCytomation, Denmark) or anti-goat HRP conjugated IgG (1:1000; DakoCytomation, Denmark), were used to detect the binding of its corresponding antibody. The protein expression was estimated with Western Lightning Chemiluminescence Reagent Plus (PerkinElmer Life Sciences, USA) and quantified by Quantity One (*version 4.6.7*) software (BioRad, USA). Membranes were stripped and re-blotted with glyceraldehyde 3-phosphate dehydrogenase antibody (anti-GAPDH, 1:10,000; Abcam, UK) and used for densitometry quantification. The raw data were analysed by normalising the relevant protein’s band intensity to the mean intensity of the housekeeping protein GAPDH. The final data were represented as arbitrary units (A.U.).

### mRNA analysis by quantitative real-time PCR

At the end of the study gastrocnemius muscle samples were harvested, placed in RNA Later (Qiagen) and stored at −80 °C. Samples (n = 10/group) were selected from each group using a random number generator and were processed for gene expression analysis. Total RNA was isolated using an RNeasy Mini kit (Qiagen) according to manufacturer’s instructions and quantified spectrophotometrically using Nanodrop 2000. RNA samples (20 ng) were subjected to quantitative real time PCR (QRT-PCR) analysis of genes of interest using the QuantiTect SYBR Green one-step RT-PCR assay (Qiagen). QRT-PCR was performed using primers for mouse *Vegf-R1* (PPM03066F), *Vegf-R2* (PPM03057A), *Trpv4* (PPM36070A), *Klf4* (PPM25088B) and *Gapdh* (QT01658692). The relative expression of these genes were calculated by using the concentration-Ct-standard curve method and normalized using the average expression of mouse *Gapdh* for each sample using the Rotor-Gene Q operating software (*version 2.0.24*) as previously reported^44,45^.

### Statistical analyses

All data were tested for normality using the D’Agostino-Pearson normality test. Data with normal distribution were expressed as mean ± standard error of mean (SEM) and analysed using parametric tests. Non-normally distributed data were expressed as median and interquartile ranges (IQR) and analysed using non-parametric tests. Statistical significance was determined using the unpaired Student t test for comparison between two groups or analysis of variance followed by Student-Newman-Keuls post-hoc analysis for comparison between multiple groups. Comparison of the time course of LDPI indices, clinical scores, open field tests and treadmill exercise tests were done by 2-way ANOVA for repeated measures, followed by Bonferroni post hoc analysis or by Linear Mixed Effect method using R Studio Software. Difference in the clinical ischemia score were determined by Fisher’s exact test. Analyses were performed using Prism (GraphPad Software, San Diego, CA) or R software. A p value of ≤*0.05* was considered to be statistically significant.

## Results

### Mice undergoing 2-stage HLI had more severe ischemia than those undergoing 1-stage HLI

Immediately after femoral artery excision, limb perfusion assessed by LDPI was similarly reduced in both HLI models by approximately 70%. Mice subjected to the 1-stage HLI had more rapid recovery of hind limb perfusion than those subjected to the 2-stage procedure (*p* = *0.014*, Fig. 1B,C, Supplementary Fig. S2). By 28 days after ischemia induction limb perfusion was similar in mice subjected to the 1-stage procedure and sham controls (*p* = *0.510*) but still reduced in mice subjected to the 2-stage procedure by comparison to sham controls (*p* < *0.001*). There was no change in overall body mass after ischemia induction (Supplementary Fig. S3).

### Mice subjected to 2-stage HLI showed more severely impaired hind limb use than those undergoing 1-stage HLI

After ischemia induction, mice subjected to both methods of HLI developed limb oedema, paleness of skin and occasional muscle necrosis. Mice in all experimental groups exhibited a severe functional deficit after surgery (Fig. 1D). Functional score was significantly worse in mice subjected to the 2-stage HLI than those subjected to the 1-stage HLI (*p* = *0.014*). Both the HLI models showed increased ischemia compared to the respective shams. There were no cases of auto-amputation or foot or limb necrosis (Supplementary Fig. S4). Mice subjected to the 2-stage HLI showed a significantly worse ischemic score compared to those subjected to the 1-stage HLI (Repeated measures 2 way ANOVA, *p* = *0.004*, Fig. 1E).

### Mice subjected to 2-stage HLI had reduced treadmill performance

**Figure 2:**
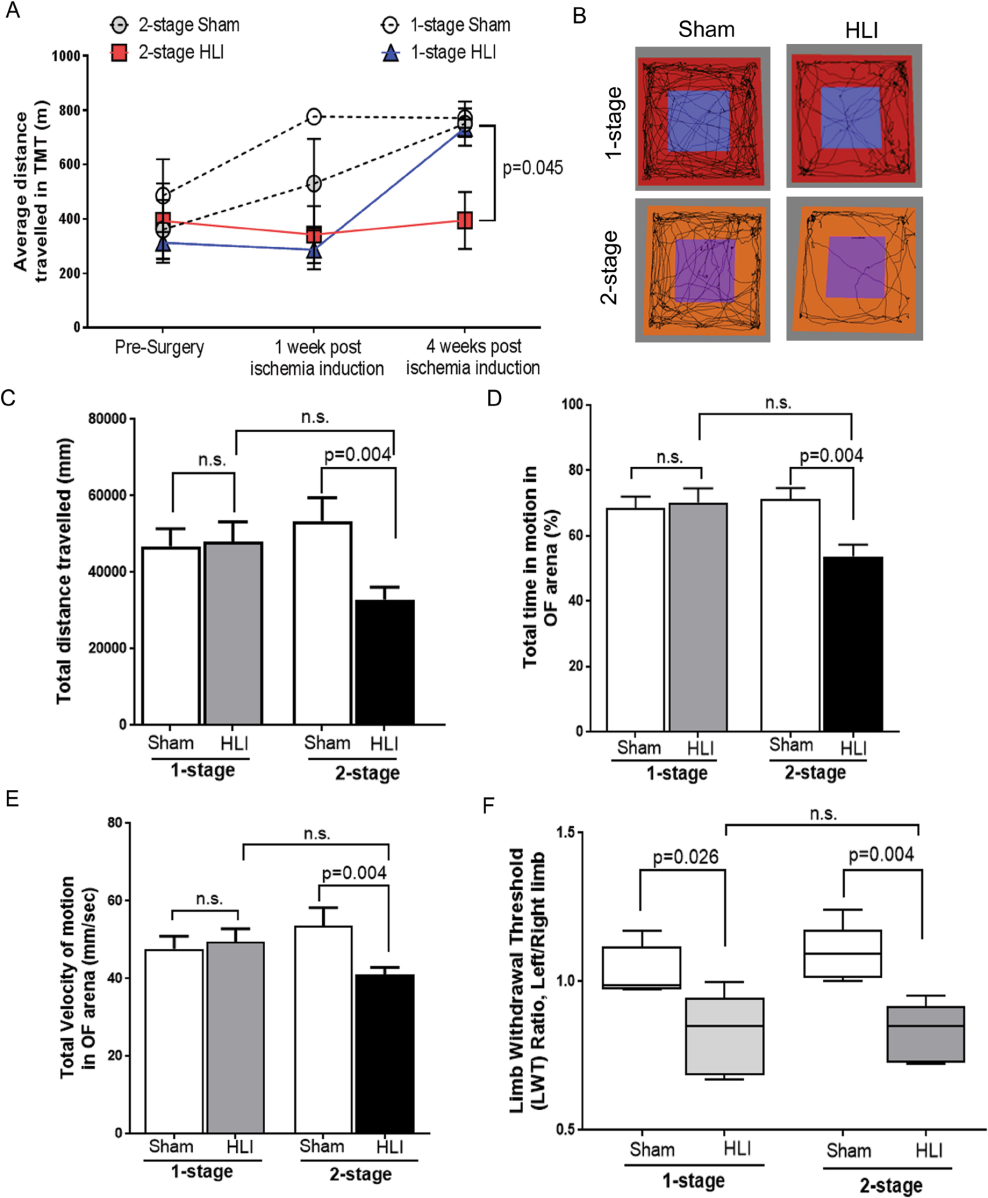
The 2-stage HLI model showed reduced ambulatory ability compared to controls. (**A**) Treadmill test (TMT) performance was reduced in the 2-stage Hind limb ischemia (HLI) model compared to the 1-stage HLI model as well as the sham controls. The 2-stage HLI group had a shorter total distance travelled 1 week after ischemia induction and by the end of the experiment period compared to sham controls. The 2-stage HLI model showed a significant reduction in total distance travelled on the treadmill compared to the 1-stage HLI group at 4 weeks. Data represented as mean ± SME, and analysed by repeated measures two-way ANOVA. (**B**) The 2-stage HLI model showed reduced voluntary activity in the Open field (OF) test. The image shows the tracking image generated by the motion tracking software. (**C**) The total distance travelled by mice subjected to 2-stage HLI on day 28 after ischemia induction were significantly less than their sham controls. (**D**) The 2-stage HLI group showed significantly lower total time in motion and (**E**), velocity of movement in the OF arena compared to their sham controls. (**F**) HLI led to impaired mechanical allodynia. The pressure sensitivities of the various experimental groups were assessed using the pain application measurement device (PAM). Assessment of mechanical allodynia by PAM showed that both the 1-stage HLI and the 2-stage HLI models had significantly increased sensitivity to pressure compared their sham controls at the end of the study period. P value significance set at ≤*0.05*.

Mice subjected to the 1-stage HLI showed no significant difference in total distance travelled during the study period on a treadmill test when compared to shams (Repeated measures 2 way ANOVA, *p* = *0.818;* Fig. 2A). After the first procedure of the 2-stage model (ameroid placement) the treadmill ambulatory performance of mice was not significantly affected (Supplementary Fig. S5A). In contrast mice subjected to 2-stage HLI had a significant reduction in total distance travelled on the treadmill compared to their sham controls (*p* = *0.050*) and mice subjected to 1-stage HLI (*p* = *0.045;* Fig. 2A).

### Mice subjected to 2-stage HLI had reduced voluntary activity

After the first procedure of the 2-stage model (ameroid placement) there was reduction in the activity of mice in the open field test (see Supplementary Fig. S5B–D). This reduction in physical activity was maintained after completing the 2-stage HLI by comparison to sham controls and also mice subjected to 1-stage HLI (Fig. 2B). The reduction in physical activity of mice subjected to 2-stage HLI was reflected in less distance travelled, less total time in motion and lower velocity of the movement compared to sham controls (Fig. 2C–E). When compared to the 1-stage HLI, the 2 stage-HLI model showed a reduction in the total distance travelled in the open field arena (Linear Mixed effect model, *p* = *0.036*).

### Mice subjected to HLI had enhanced mechanical allodynia

Mice subjected to both 1-stage and 2-stage HLI showed significantly increased sensitivity to pressure compared their respective sham controls and there was no significant difference in pressure sensitivity between the two models (Fig. 2F).

### HLI induces systemic inflammation

The plasma concentrations of the cytokines assessed were below the detectable ranges in both the sham control groups (Table 1). Mice subjected to HLI, irrespective of model, had plasma cytokine concentration significantly higher than the sham controls although levels were not significantly different between models (Table 1). The plasma concentrations of nitric oxide (NO) metabolites were higher in mice undergoing 1-stage HLI than the sham control group (*p* < *0.001*) and mice undergoing 2-stage HLI (*p* = *0.031*, Table 1).

**Table 1:** The concentrations of cytokines (pg/ml) in the plasma of the 1 stage and 2 stage HLI models.

Proteins	1 stage -Sham (N = 6)	1 stage HLI (N = 10)	2 stage -Sham (N = 6)	2 stage HLI (N = 10)	*P value*
Monocyte Chemoattractant Protein-1 (MCP-1)	ND	39.28 ± 11.77	ND	30.33 ± 20.64	*0.697*
Interleukin-6 (IL-6)	ND	4.12 ± 1.48	ND	2.36 ± 0.58	*0.334*
Interleukin-10 (IL-10)	ND	11.81 ± 6.06	ND	8.35 ± 4.27	*0.690*
Interferon-γ (IFN-γ)	ND	2.62 ± 1.22	ND	0.52 ± 0.34	*0.233*
Tumor Necrosis Factor (TNF)	ND	5.45 ± 2.93	ND	3.17 ± 1.50	*0.591*
Interleukin-12p70 (IL-12p70)	ND	21.60 ± 9.62	ND	46.05 ± 25.56	0.825
NO (nitrite + nitrate)	11.20 ± 2.17	61.18 ± 10.50^$^	21.13 ± 6.07	42.98 ± 20.33	0.031^*^

The concentrations of cytokines (pg/ml) were measured in the platelet poor plasma (n = 6–10/group) collected at the end of the study using a cytometric bead assay. NO metabolites were assessed by the Griess assay. Data expressed as mean ± standard error of mean (SEM) and analysed by student’s t-test. P values are for two-way comparisons between the 1-stage and 2-stage HLI models. Statistical significance considered as P value ≤0.05. (*)  = ≤0.05, 2-stage HLI compared to 1-stage HLI; ($)  = <0.001, 1-stage HLI compared to shams. Abbreviations: IL, Interleukin; IFN, Interferon; MCP, Monocyte Chemoattractant Protein; NO, total nitrite and nitrate metabolites; TNF; Tumour Necrosis Factor.

### Mice subjected to 2-stage HLI had increased gastrocnemius muscle inflammation and fibrosis

**Figure 3:**
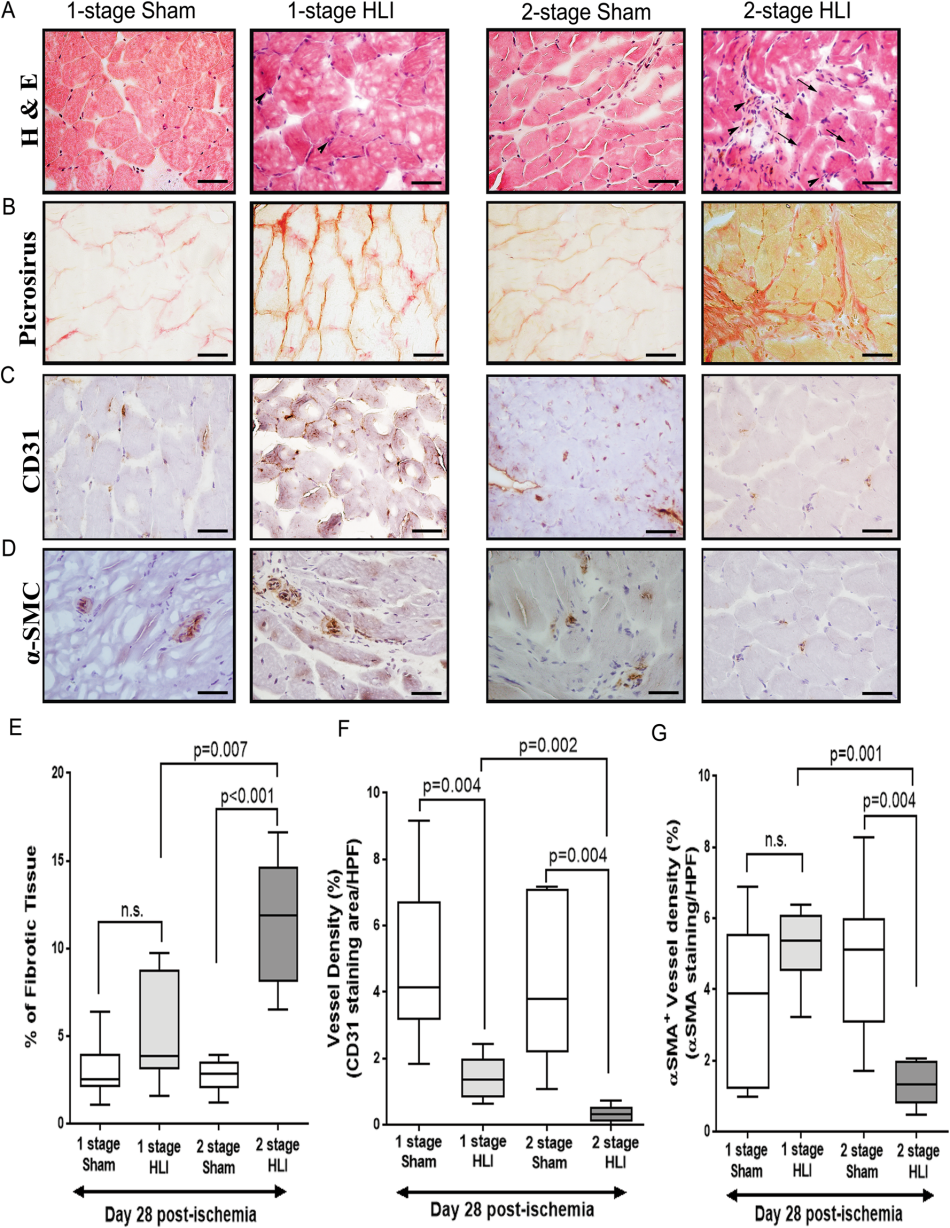
The 2 stage HLI model showed increased fibrosis and reduced angiogenesis compared to the 1-stage HLI model. (**A**) Representative hematoloxylin and eosin (H&E) staining of gastrocnemius muscle sections from the 1-stage and 2-stage Hind limb ischemia (HLI) models and respective sham controls at day 28. Normal morphology in the gastrocnemius sections of sham controls was evidenced by intense eosin staining and the presence of peripheral nuclei. Arrows and arrowheads indicate regeneration and remodelling of muscle bundles and invasion of inflammation of inflammatory cells, respectively. Persistent inflammation is evident in samples from the 2-stage HLI model taken at day 28 (arrow heads). (**B**) Fibrotic changes in gastrocnemius muscle after femoral artery ligation. Representative Picrosirius red-stained histological sections showing increased fibrotic (red) content indicating significant increase of interstitial fibrosis in the ischemic limb. (**C**) Representative immunohistochemical images showing neovascularisation after HLI in the two models assessed by CD31-positive staining (dark brown staining). The panel shows images from the ischemic limbs from the 1-stage and 2-stage HLI models and the respective sham controls (n = 8/group). (**D**) Representative immunohistochemical images showing α-Smooth muscle actin (α-SMA)–positive vessel density (dark brown staining). The panel shows the data from the ischemic limbs from the 1-stage and 2-stage HLI models and the respective sham controls (n = 8/group, scale bars in all images  = 25 µm). (**E–G**) Quantitative bar graphs showing the effect of HLI on (**E)** skeletal muscle fibrosis by picrosirius red staining, **(F)** angiogenesis by immunohistochemical staining against CD31 and **(G)** arteriogenesis by immunohistochemical staining against α-SMA. All values are median and interquartile ranges (n = 8/group) and p value significance set at ≤*0.05*.

Myofibers which are healthy and functionally active are characterised by peripheral nuclei, while myofibrils with central nuclei are immature and do not show optimal contraction^46^. In gastrocnemius muscle samples removed from sham controls, myocytes were angular with peripheral nucleus (Fig. 3A). At day 28 after ischemia induction, mice subjected to 2-stage HLI showed microscopic changes such as cellular swelling, focal necrosis and interstitial oedema. There were also numerous infiltrating inflammatory cells. Gastrocnemius muscle samples from mice subjected to 1-stage HLI showed more homogenous appearance with all myocytes showing peripheral nuclei and limited inflammatory cell infiltration (Fig. 3A). Histological evaluation revealed that tissues of mice subjected to 1-stage HLI had fewer immature myofibers cells than the tissues from 2-stage HLI model. Furthermore, muscle samples from mice subjected to 2-stage had prominent oedema, myofibre separation and multifocal neutrophilic infiltration. Neutrophils were observed throughout the tissue sections. Necrotic muscle fibres were prominent and formed confluent areas (Fig. 3A). Muscle fibrosis was assessed by picrosirius red staining which suggested that 2-stage HLI led to increased skeletal muscle fibrosis compared to 1-stage HLI (*p* = *0.007*) or sham controls (*p* = *0.001*; Fig. 3B,E).

### Mice subjected to 2-stage HLI had fewer hind limb collaterals

Consistent with the reduced perfusion as assessed by LDPI, both arteriogenesis and angiogenesis was inhibited in the ischemic gastrocnemius muscles of the 2-stage HLI model (Fig. 3C,D**)**. Measurement of angiogenesis by CD31 immunostaining showed that the presence of arterioles was significantly reduced in samples from the 2-stage HLI compared to the 1-stage HLI (*p* = *0.002*) or sham controls (*p* = *0.004*; Fig. 3). The arteriolar density was also significantly less in samples from the 2-stage HLI model compared to the 1-stage HLI (*p* = *0.001*) or sham control (*p* = *0.004*; Fig. 3).

### Protein concentrations of angiogenesis and shear stress response markers were downregulated in the gastrocnemius muscles of mice undergoing 2-stage HLI

**Figure 4:**
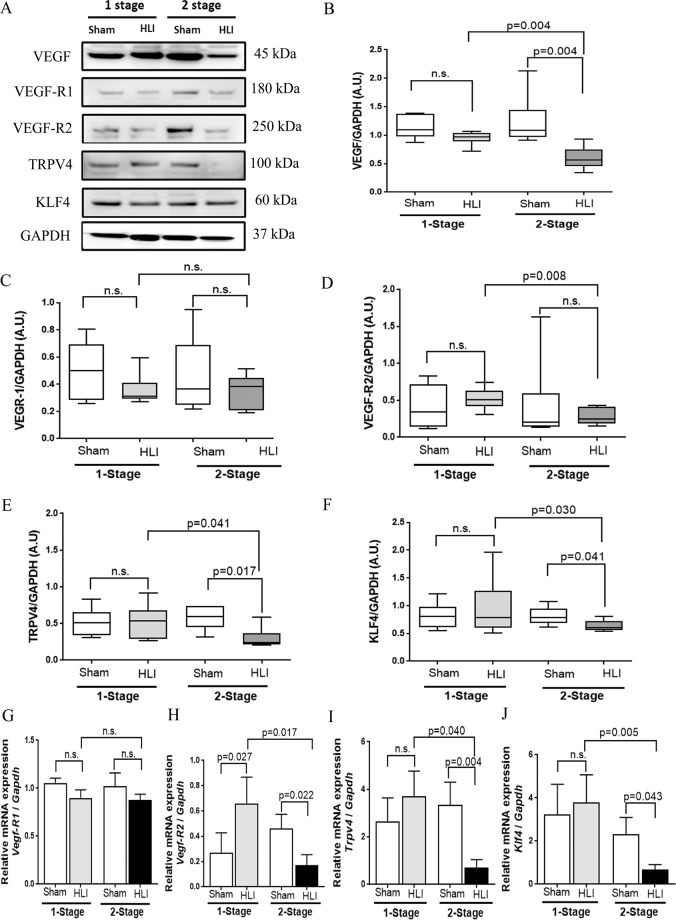
Comparison of protein and gene expression within the gastrocnemius muscles of mice undergoing the 1-stage and 2-stage HLI. (**A**) Representative Western blot images showing the expression levels of various proteins within the gastrocnemius muscles of mice undergoing Hind limb ischemia (HLI). (**B**) Quantitative graph showing that total Vascular endothelial growth factor (VEGF) protein levels were significantly downregulated in the 2-stage HLI model compared to sham controls and the 1-stage HLI model. (**C**) Quantitative graph showing no change in expression of Vascular endothelial growth factor receptor (VEGFR)-1 in response to HLI. (**D**) Quantitative graph showing reduced expression of VEGFR-2 in the 2-stage HLI model compared to the 1-stage model. (**E–F**) Quantitative graph showing that the fluid shear stress responder proteins Transient receptor potential cation channel subfamily V member 4 (TRPV4) (**E**) and Kruppel Like Factor 4 (KLF4) (**F**) were both significantly downregulated in the 2-stage model compared to sham controls and the 1-stage model. Data expressed as median and interquartile ranges with maximum and minimum data points (whiskers) and compared by Mann-Whitney U test (n = 6–8/group). (**G–J**) Relative gene expressions of *Vegf-R1* **(G)**, *Vegf-R2* **(H)**, *Trpv4* **(I**) and *Klf4* **(J)** assessed in the gastrocnemius muscles of mice undergoing HLI. Quantitative real time PCR (QRT PCR) was performed on extracted total mRNA using specific primers and normalised to glyceraldehyde 3 phosphate dehydrogenase *(Gapdh)* expression. Data analysed by Mann-Whitney U test (n = 10 samples/group).

The relative total VEGF and VEGFR-2 (but not VEGFR-1, p-eNOS/eNOS and HIF-α) protein levels in gastrocnemius tissue collected from mice 4 weeks after 2-stage HLI were significantly less than in sham controls and mice undergoing 1-stage HLI (Fig. 4A–D, Supplementary Fig. S6). Analysis of tissues from the 2-stage HLI model prior to ameroid placement and day 3 and day 7 after ameroid placement suggested no significant changes in concentrations of TRPV4 or VEGF in response to ameroid constriction (Supplementary Fig. S7). At the end of the experiment, protein concentrations of TRPV4 and KLF4 were significantly less in the gastrocnemius muscle samples from mice undergoing 2-stage HLI compared to sham controls and mice undergoing 1-stage HLI (Fig. 4E,F). QRT-PCR showed that the relative expressions of *Vegf-R2, Trpv4* and *Klf4* in gastrocnemius muscle of mice subjected to 2-stage HLI were significantly lower than within the gastrocnemius muscle of mice undergoing 1-stage HLI or in sham controls (Fig. 4G–J).

### Exercise training improved functional capacity but not limb perfusion in mice subjected to 2-stage HLI

**Figure 5:**
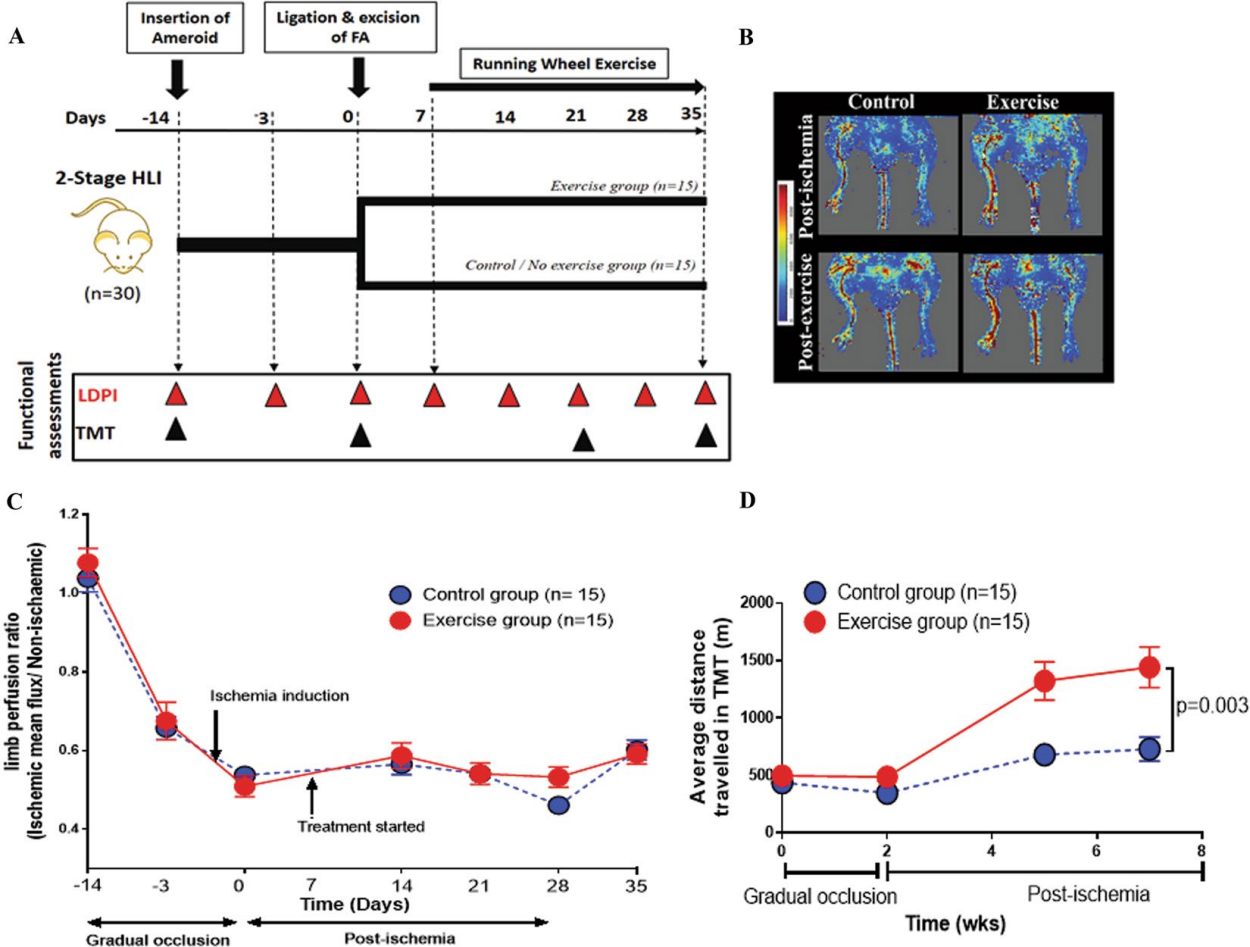
Exercise training led to increased ambulatory ability of mice undergoing 2-stage HLI. (**A**) Schematic representation of the design of the experiment. (**B**) Examples of Laser Doppler perfusion imaging (LDPI) images showing the relative perfusion of the limbs of mice in the control (**C**) and exercise groups (**E**). **(C**) Quantitative graph showing no significant difference in the perfusion of the hind limbs of mice after exercise training in comparison to controls. Data analysed by linear mixed effect analysis. (**D**) The average distance travelled on the treadmill was significantly greater in mice receiving exercise training than sedentary controls. Data analysed by repeated measures 2-way ANOVA.

Supervised exercise training is an established method to improve functional capacity in PAD patients^47,48^ and previous studies show that mice respond positively to exercise training^49,50^. In order to examine whether an established clinically effective therapy was effective in the novel animal model, the effect of exercise training (using a running wheel) on functional capacity of mice subjected to 2-stage HLI was assessed. Exercise training was commenced 5 days after ischemia induction. Exercise training did not affect limb perfusion as assessed by LDPI (Linear Mixed effect test, *p* = *0.700*; Fig. 5B,C). Mice subjected to exercise training showed a significant increase in average treadmill walking capacity compared to sedentary controls (*p* = *0.003*; Fig. 5D).

### Exercise training upregulated gastrocnemius muscle VEGF and TRPV4 levels

**Figure 6:**
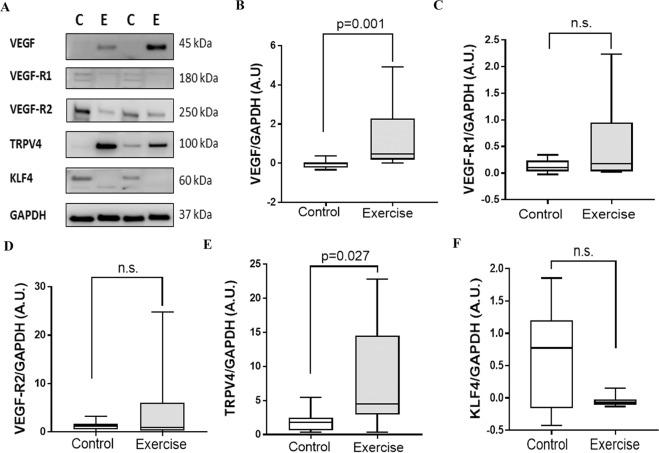
Exercise training led to upregulation of gastrocnemius muscle VEGF and TRPV4 in the 2-stage HLI model. (**A**) Representative Western blot images showing the expression levels of various proteins assessed in the gastrocnemius muscle of mice after exercise training. (**B**) Quantitative graph showing that total Vascular endothelial growth factor (VEGF) protein levels were significantly upregulated in the exercise training group compared to the sedentary controls. (**C**,**D**) Quantitative graphs suggesting that the levels of Vascular endothelial growth factor receptor (VEGFR)-1 and VEGFR-2 did not show any significant changes in response to exercise training. (**E**) Protein levels of fluid shear stress responder Transient receptor potential cation channel subfamily V member 4 (TRPV4) was greater in the exercise training group compared to the controls. (**F**) Total protein levels of Kruppel Like Factor 4 (KLF4) were similar in mice undergoing exercise training and sedentary controls. Data expressed as median and interquartile range with maximum and minimum data points (whiskers) and compared by Mann-Whitney U test (n = 6–8/group).

Since improvement in ambulatory performance as a result of exercise training could be due to enhanced angiogenesis, the expression of angiogenesis and shear stress responsive proteins VEGF, VEGF-R1, VEGF-R2, TRPV4 and KLF4 were assessed in the gastrocnemius muscles (Fig. 6**)**. VEGF (*p* = *0.001*, Fig. 6B) and TRPV4 (*p* = *0.027*, Fig. 6E) but not VEGF-R1, VEGF-R2 and KLF4, were highly upregulated following exercise training (Fig. 6C,D,F).

## Discussion

This report describes the development of a novel model of HLI which results in more severe and prolonged ischemia than the traditional model. Mice subjected to the 2-stage HLI had functional and ambulatory impairment and a positive response to exercise training as has been reported for PAD patients^51^.

Previous rodent studies suggest that placing of ameroid constrictors alone without manipulating the femoral artery results in mild ischemia^20^ and blood flow recovers within 2 weeks^16^. Hence, the novel HLI model was based on placement of two ameroid constrictors on the femoral artery to promote gradual occlusion followed by excision of intervening segment after 14 days to induce severe ischemia. A previous report suggests that recovery of hind limb blood flow is reduced in *ApoE*^*−/−*^ compared to C57BL/6 J mice due to their limited collateral arteries and hence *ApoE*^*−/−*^ mice were used in the current study^24^. PAD patients are generally older and exhibit metabolic derangements that limit angio- and arterio-genesis and previous studies suggest that *ApoE*^*−/−*^ mice have delayed skeletal muscle healing^52^, reduced angiogenesis responses and impaired functional recovery after HLI further supporting the rationale for choosing this mice species^26,28,53^.

Mice subjected to 1-stage HLI had rapid recovery of limb blood flow and reached a perfusion level similar to the sham controls within 28 days as has been reported by other investigators^28,54,55^. Ligation and sudden excision of the femoral artery is believed to generate a pressure gradient between the proximal and distal ends of the occluded vessel, resulting in increased shear stress and a redirection of blood flow towards the collaterals and through numerous branches arising from the internal iliac artery, resulting in rapid improvement in blood flow^14,56^. Ameroid constrictors have been shown to cause luminal occlusion within 14 days, however, the blood flow slowly increases in the next 2–3 weeks^16,21^. Hence, in the new model, we superimposed a secondary acute event by excising the intervening femoral artery segment along with the ameroid constrictors. In contrast to the 1-stage HLI, mice subjected to 2-stage HLI had on-going limb ischemia and a prolonged functional deficit on both forced and voluntary ambulation tests. These findings support the value of the novel model for the testing of interventions aimed at achieving clinical improvements in PAD patients.

Since angiogenesis is an inflammation-driven process^57^, the concentrations of circulating cytokines were measured. These markers of systemic inflammation increased in response to HLI in both models examined. Twenty eight days after HLI induction, the concentrations of circulating cytokines were similar in the two models studied. Gastrocnemius muscle samples obtained from the 2-stage HLI model had marked neutrophilic infiltration. It has been previously reported that inflammatory cells accumulate in hypoxic tissues and promote angiogenesis^58^. It is possible that the systemic concentrations of cytokines were not reflective of the level of inflammation within the hind limb. Markers of angiogenesis and arteriogenesis in gastrocnemius tissue, such as CD31 and α-SMC, were found to be less evident in the 2-stage than the 1-stage HLI model. Furthermore, gastrocnemius VEGF and VEGF-R2 protein levels and amount of total plasma NO metabolites were significantly lower in the 2-stage than 1-stage HLI model. VEGF promotes angiogenesis^59^ through binding to VEGF-R2^60^ expressed on endothelial cells^61^. VEGF induces the release of NO^62^ thereby promoting microvascular perfusion and endothelial progenitor cell mobilization^63–65^. Endothelial cell derived microRNA, such as miR-16, have also been implicated in controlling angiogenesis through inhibiting Rho GDP dissociation inhibitor (RhoGDI)-α, an important regulator of eNOS phosphorylation^66^. It appears likely that the low levels of pro-angiogenic markers in the 2-stage HLI model reflect less activation of endothelium-dependent pro-angiogenesis signalling pathways which were stimulated by collateral flow within the 1-stage model.

Shear stress promotes arteriogenesis by stimulating remodelling of collaterals^67,68^. Endothelial cells transduce changes in shear stress into intracellular signals which promote expression of a distinct set of genes which can control the response to ischemia^69–71^. Previous studies suggest that increased shear stress promotes phosphorylation and upregulation of mechano-sensors, such as TRPV4^72–75^. It was postulated that the distinct ways of inducing HLI in the two models studied might be reflected in different TRPV4 expression. Mice subjected to 2-stage HLI had lower expression of TRPV4 compared to those undergoing 1-stage HLI. Furthermore, in the 2-stage HLI model, there was no change in TRPV4 protein levels after ameroid placement, suggesting that ameroid constriction is a gradual process that does not lead to shear stress changes capable of stimulating mechano-sensors like TRPV4. These findings also suggest that 2-stage HLI results in more limited collateral flow than the 1-stage approach. The acute reduction in arterial pressure gradient following femoral artery excision in the 1-stage HLI model it thought to be registered by endothelial shear stress response elements resulting in upregulation of angiogenesis and arteriogenesis promoting genes^21^. Simulation of TRPV4, for example by 4α-phorbol 12, 13-didecanoate, has been reported to promote increase NO release and increased hind limb blood flow^73^. TRPV4 deficient rodents have impaired vasodilatation^76–79^. This supports the important role of TRPV4 in promoting adaptation to acute limb ischemia.

Four weeks of exercise training led to an approximate 2-fold increase in treadmill ambulatory distance in the mice subjected to 2-stage HLI, paralleling findings from PAD patients^80–83^. These findings suggest the novel 2-stage HLI mouse model simulates the walking impairment experienced by patients. In support of the relevance of this model to patients, we found that exercise therapy improved treadmill performance without improving limb perfusion, a finding similar to that described in PAD patients^47,48,84^. Since exercise training increases shear stress in pre-existing collaterals we examined the expression of TRPV4 and angiogenesis markers^85^. Compared to sedentary controls, mice undergoing exercise training showed increased total protein levels of VEGF and TRPV4, which was in accordance with previous reports showing enhanced expression of pro-angiogenesis markers after exercise training^86–88^. Overall these findings suggest flow-mediated upregulation of shear stress responsive genes is important in stimulating angiogenesis responses in the new model.

This study have several strengths and weaknesses. The 1-stage HLI model which is usually utilised for PAD research has many limitations including its disparate pathophysiological mechanisms compared to patient presentations, the temporary nature of the ischemia and its relative responsiveness to a variety of therapies which are not effective in patients^14^. This study suggests the novel 2-stage model has clear advantages over the 1-stage model since ischemia is more severe and prolonged and does not naturally recover making it suitable to access the longer term effects of potential therapies. The study also has some limitations. These include the absence of a detailed time course of the genes and proteins assessed and the absence of a definitive test of the role of shear stress response elements in the differences in the models found. The importance of shear stress response elements in the response to HLI has however been previously reported in prior studies^85^. It is therefore logical to assume similar mechanisms were involved in the current study given the gene expression differences demonstrated. The study also lacked the use of new high resolution imaging systems, such as fluorescent microsphere angiography.

### Clinical perspectives

(i) Many findings within currently used animal models of peripheral arterial disease (PAD) have not translated to patients hence a clinically relevant animal model of PAD is needed.
(ii) HLI was induced in male Apolipoprotein E deficient mice by a 2-stage procedure of initial gradual femoral artery occlusion by ameroids and subsequent excision of the intervening segment of the femoral artery.
(iii) This novel preclinical model showed more severe and sustained ischemia than the traditional model and may be useful in the evaluation of potential PAD therapies.

## Conclusion

This study suggests that the ischemic characteristics and molecular mechanisms underlying the acute 1-stage HLI and the novel 2-stage HLI models are distinct. Most importantly the novel 2-stage model illustrates clinically-relevant characteristics, such as persistent and severe ischemia, suggesting it may be valuable for pre-clinical testing of potential angiogenesis therapies.

## Supplementary information

Supplementary Information.
