# Supplementary material for: Development of a two-stage limb ischemia model to better simulate human peripheral artery disease
Source: Sci Rep. 2020 Feb 26;10:3449. doi: 10.1038/s41598-020-60352-4 (PMC7044206; doi:10.1038/s41598-020-60352-4)

SUPPLEMENTAL MATERIAL

Development of a two-stage limb ischemia model to better simulate human peripheral artery disease

Running title: Novel animal model of peripheral artery disease

Smriti M Krishna. M.Phil., Ph.D.[1], Safraz Mohamed Omer, PhD[1], Jiaze Li, Ph.D.[1], Susan K Morton, Ph.D.[1], Roby J Jose, Ph.D.[1], Jonathan Golledge, MB BChir, MA MChir, FRCS, FRACS [1, 2].

1. The Vascular Biology Unit, Queensland Research Centre for Peripheral Vascular Disease, College of Medicine and Dentistry, James Cook University, Townsville, Queensland, Australia 4811.

2. Department of Vascular and Endovascular Surgery, The Townsville Hospital, Townsville, Queensland, Australia 4811.

Correspondence and reprint requests to:

Professor Jonathan Golledge
The Vascular Biology Unit,
Queensland Research Centre for Peripheral Vascular Disease,
James Cook University,
Townsville, Queensland,
Australia 4811
Tel: +61 7 4781 4730
Email: jonathan.golledge@jcu.edu.au

Supplementary Tables

Table S1

Descriptors for the Tarlov functional scoring based on the use of the ischemic versus non-ischemic contralateral limb.

Score	Description
0	No movement
1	Barely perceptible movement, no weight bearing
2	Frequent and vigorous movement, no weight bearing
3	Supports weight, may take 1 or 2 steps
4	Walks with only mild deficit
5	Normal but slow walking
6	Full and fast walking

Table S2

Descriptors for ischemia scoring based on the appearance of the ischemic versus non-ischemic contralateral limb.

Score	Description
0	Auto-amputation of leg
1	Leg necrosis
2	Foot necrosis
3	Two or more toe discoloration
4	One toe discoloration
5	Two or more nail discolorations
6	One nail discoloration
7	No necrosis

Supplemental Figures

Supplemental Figure 1

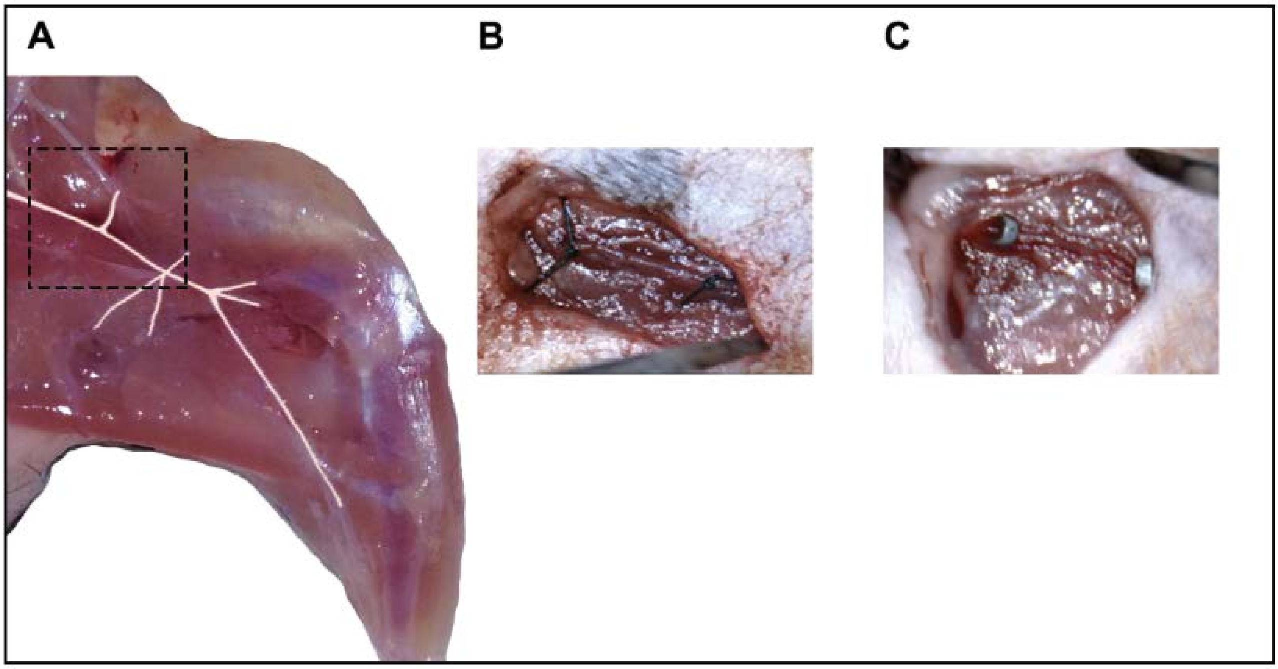

Figure S1. **Schema of mouse hind limb ischemic (HLI) model**s. The two HLI models were developed in male Apolipoprotein E deficient mice. **A**, shows the region where the surgical incision and manipulations of femoral artery was performed. The study utilised the most commonly used unilateral acute HLI model (**B**, 1-stage HLI) and the newly developed 2-stage HLI model (**C**).

Supplemental Figure 2

A.

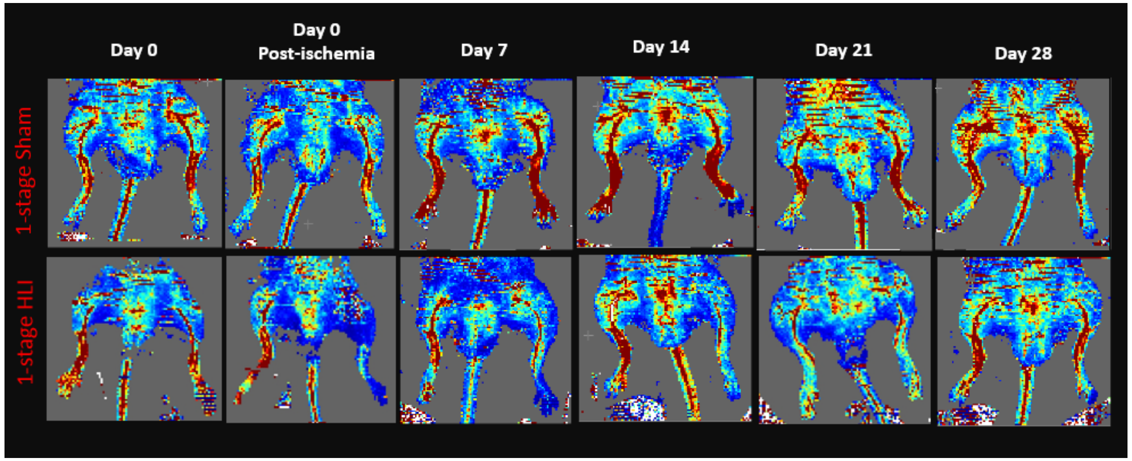

B.

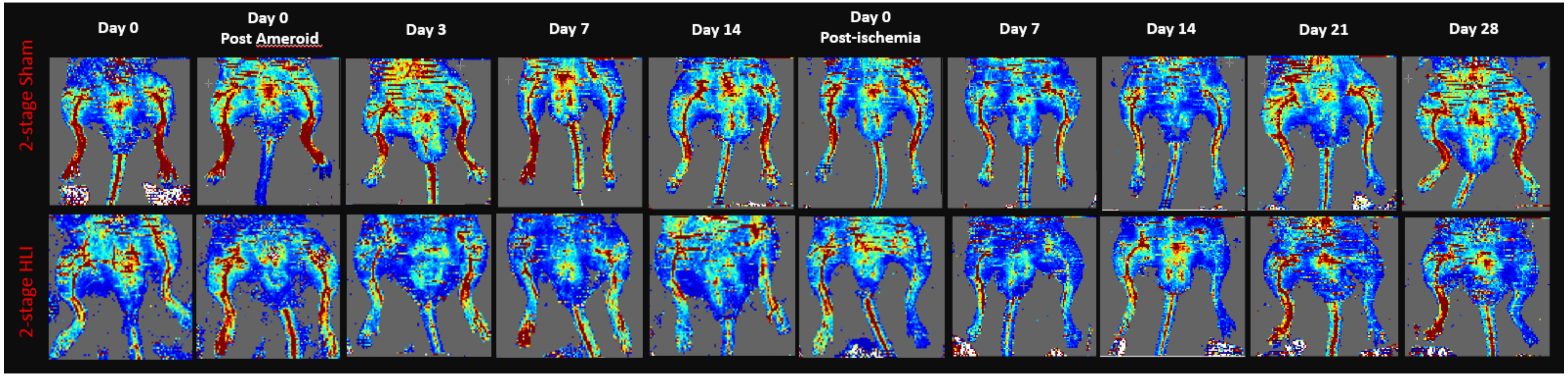

Figure S2. Representative images showinghind limb blood flow in the 1-stage and 2-stage HLI models and their respective sham groups monitored *in vivo* by laser Doppler perfusion imaging (LDPI). In the color-coded images, normal baseline (control) perfusion in both hind limbs is depicted in red. Immediately after unilateral femoral artery excision marked reduction in blood flow of one hind limb is depicted in blue.

Supplemental Figure 3

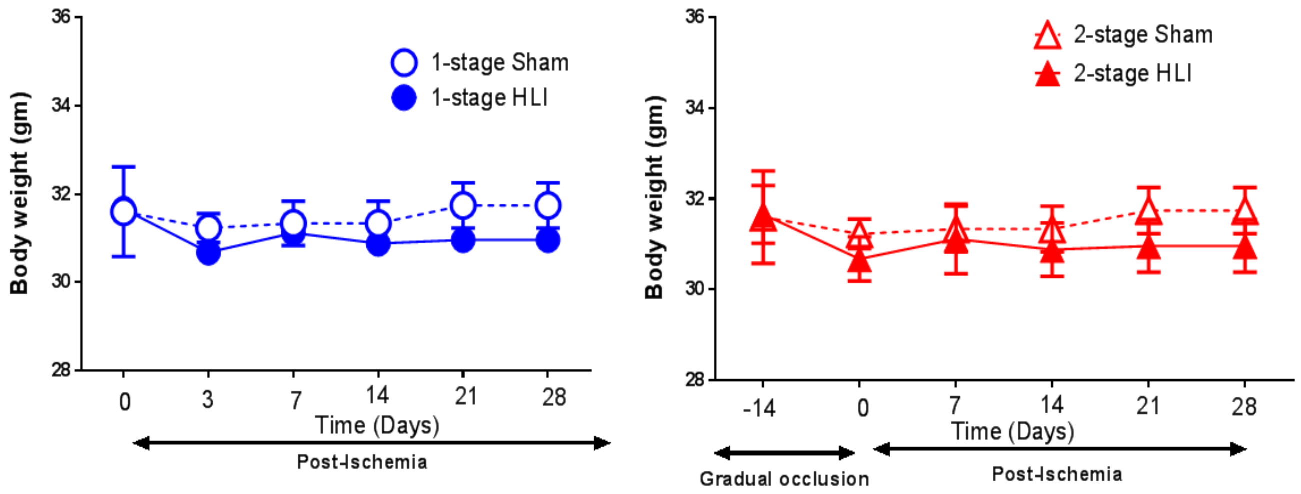

Figure S3. Graphs showing the total body weight of the two hind limb ischemia (HLI) experimental groups and the respective sham controls throughout the study period.

Supplemental Figure 4

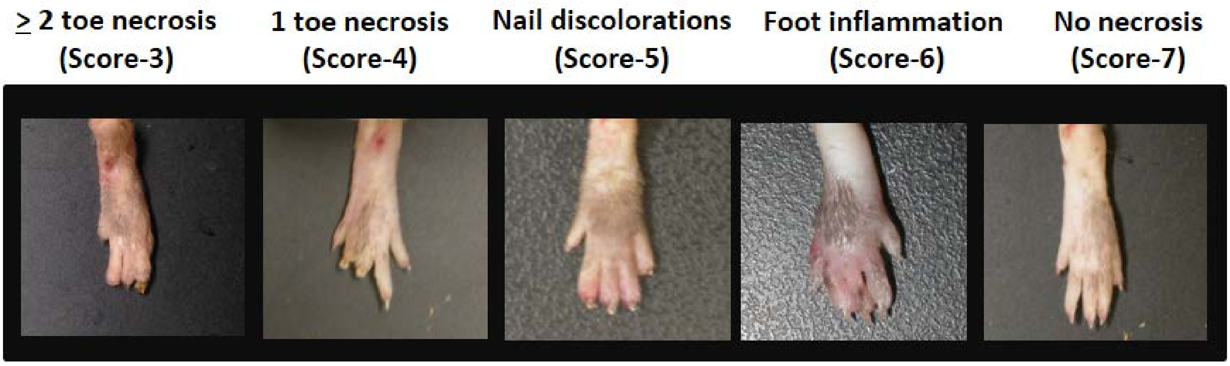

Figure S4. Descriptors for scoring the appearance of the ischemic versus non-ischemic contralateral limb.

Supplemental Figure 5

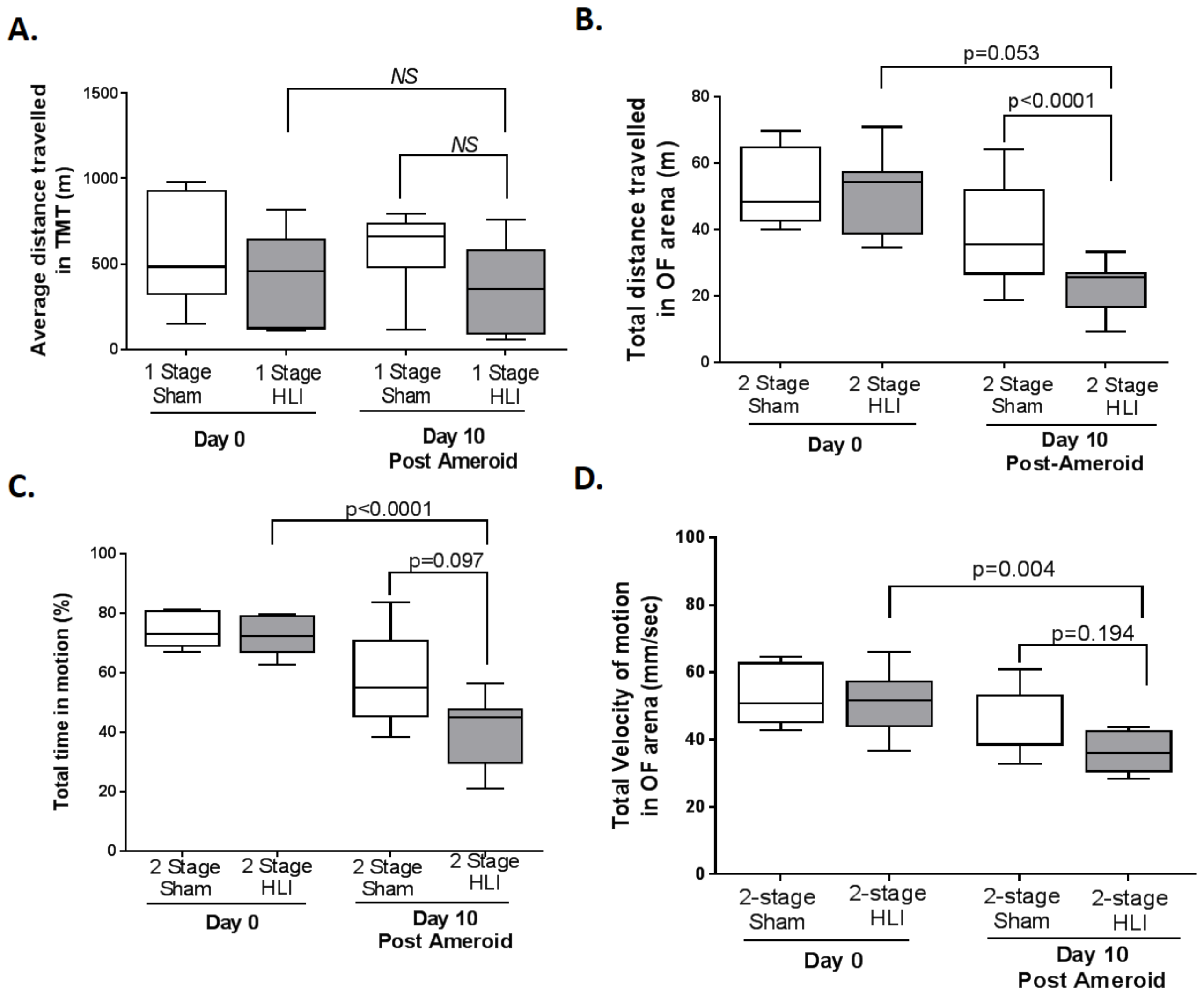

Figure S5. Quantitative graphs showing; **A.** The average distance travelled in a TMT after ameroid insertion. **B.** Graph showing the total distance travelled inside the OF arena after ameroid insertion. **C.** Graph showing the total time in motion in the OF arena by the two experimental groups and their respective sham controls. **D.** Graph showing the total velocity of motion in the OF arena. *Abbreviations:* TMT, Treadmill Test; OF, Open field; HLI, Hind limb ischemia.

Supplemental Figure 6

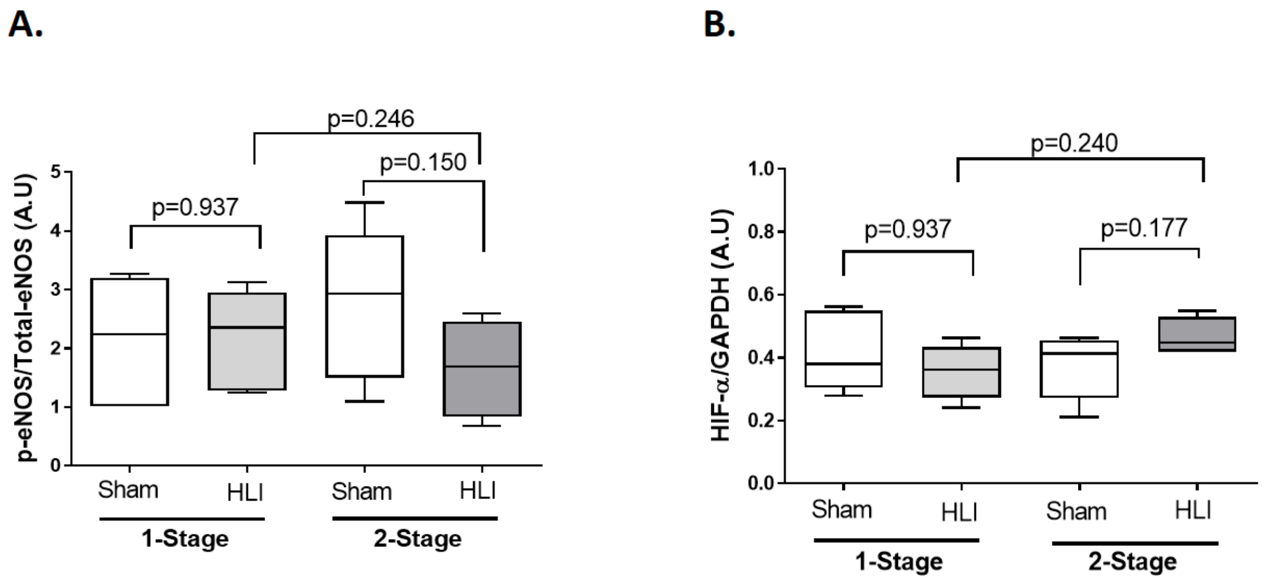

Figure S6. Quantitative western blot analysis of proteins analysed in the ischemic muscle tissues at the end of the study. *Abbreviations:* A.U, Arbitrary units; eNOS, endothelial Nitric Oxide; p-eNOS, phosphorylated endothelial Nitric Oxide; GAPDH, glyceraldehyde 3 phosphate dehydrogenase; HIF, Hypoxia induced factor; HLI, Hind limb ischemia.

Supplemental Figure 7

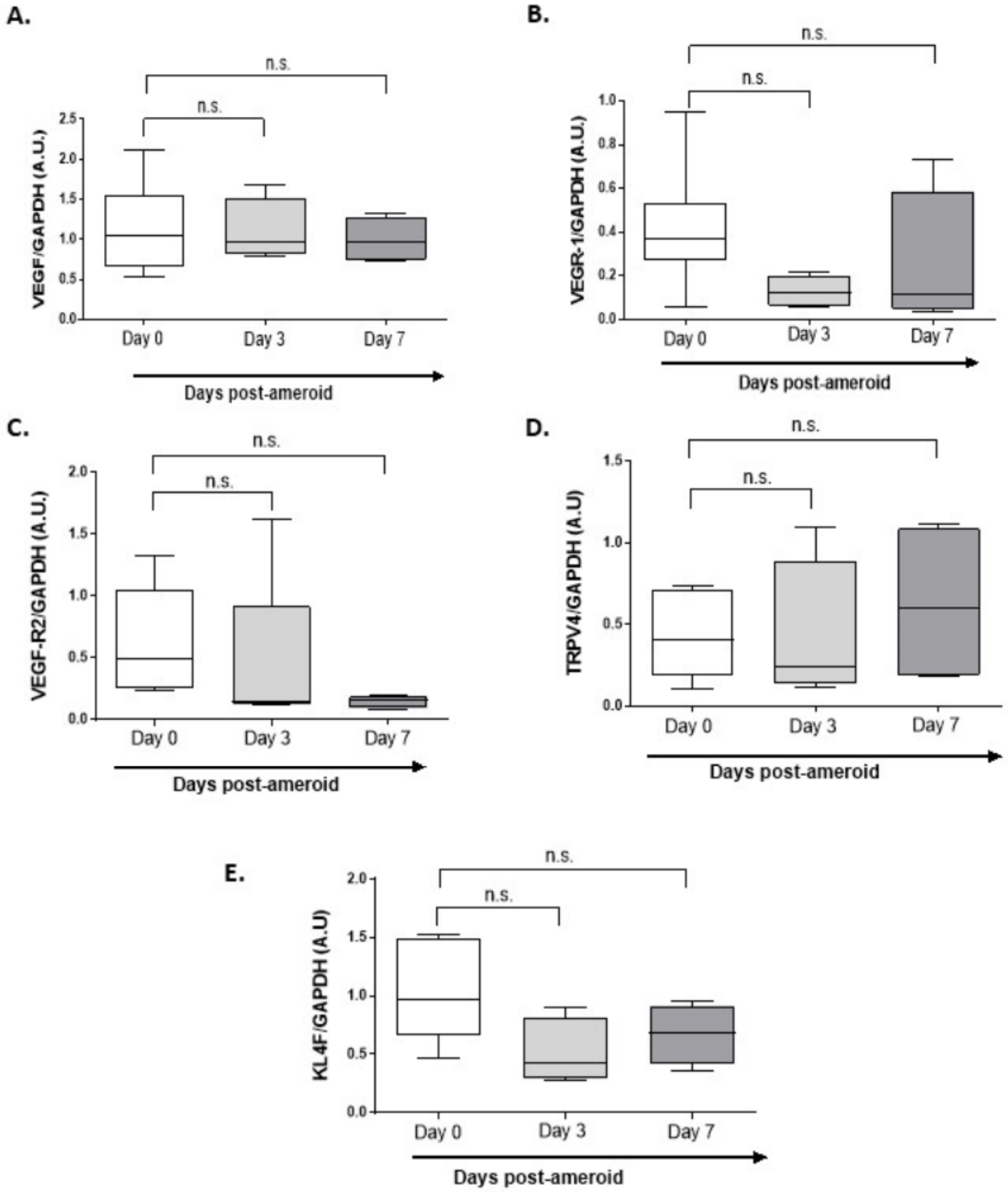

Figure S7. Quantitative Western blot analysis of proteins analysed in the gastrocnemius muscle tissues in the 2-stage HLI model. Tissue were harvested prior to and days 3 and 7 after ameroid placement. Data analysed by one-way ANOVA. *Abbreviations:* A.U, Arbitrary units; GAPDH, glyceraldehyde 3 phosphate dehydrogenase; HLI, Hind limb ischemia; KLF4, Kruppel Like Factor 4; TRPV4, Transient receptor potential cation channel subfamily V member 4; VEGF, Vascular endothelial growth factor; VEGF-R, Vascular endothelial growth factor receptor.

Supplementary Data- Un-edited gel pictures

Full un-edited gels of Figure 4 A.

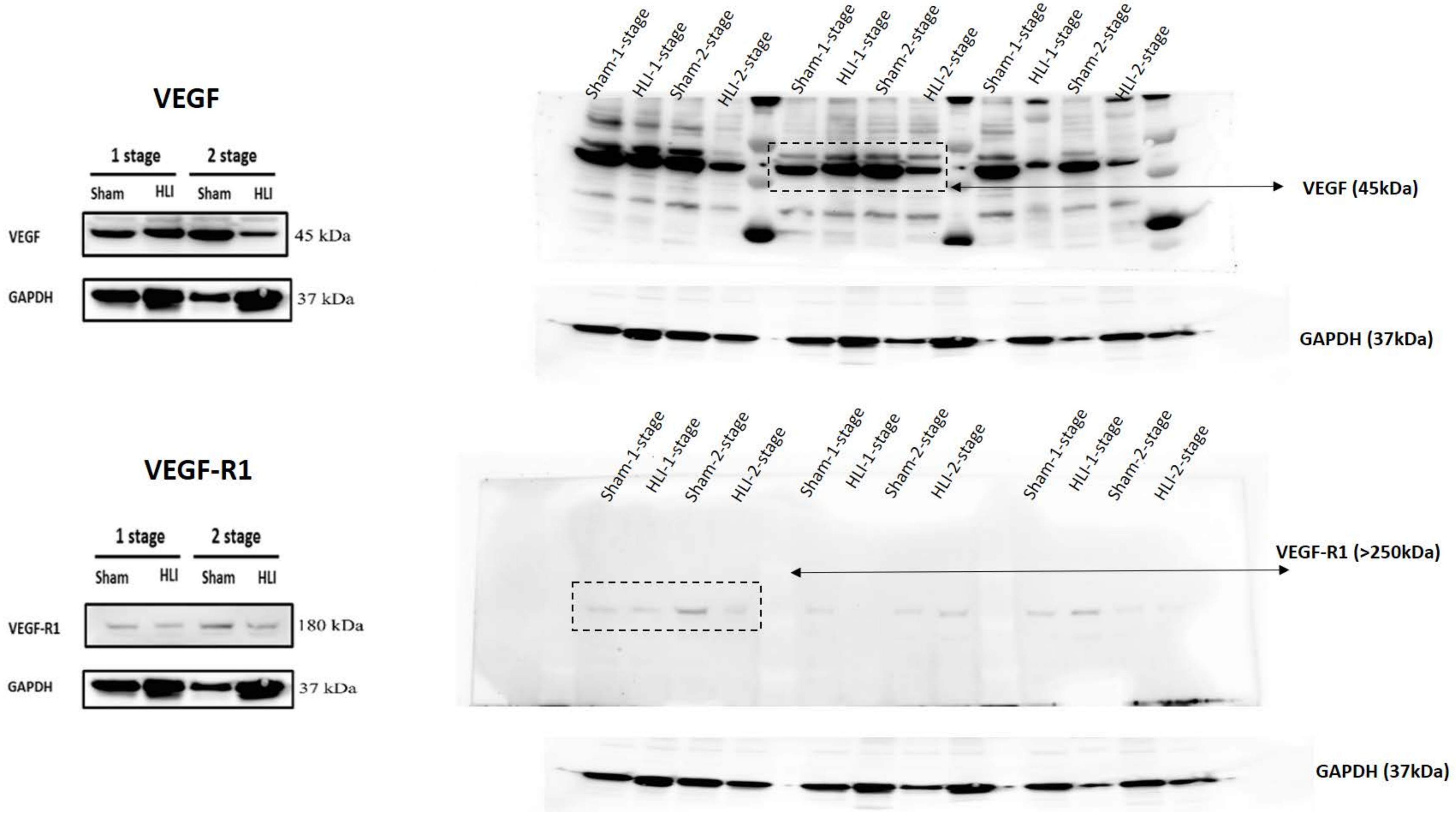

Full un-edited gels of Figure 4 A.

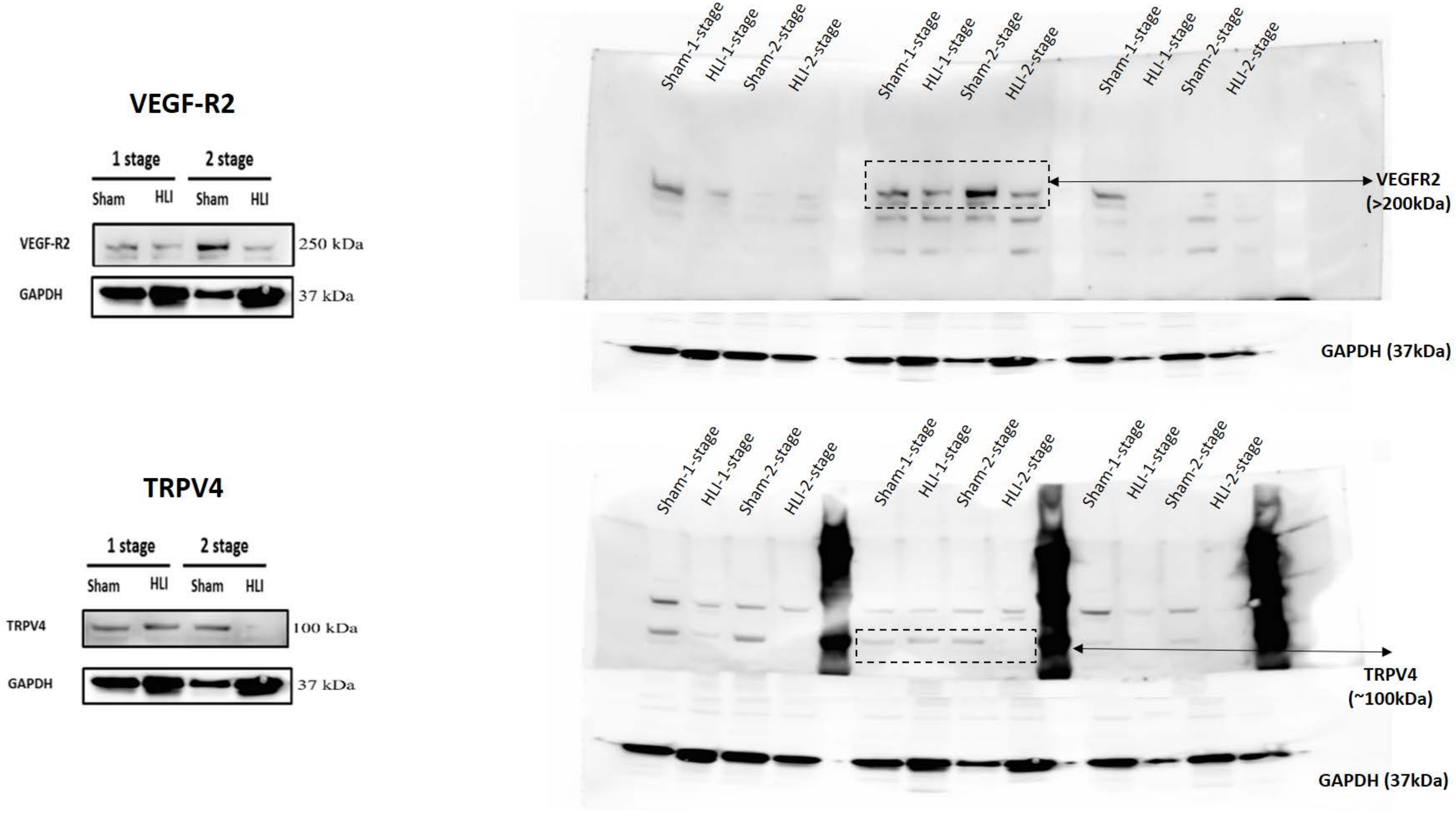

Full un-edited gels of Figure 4 A.

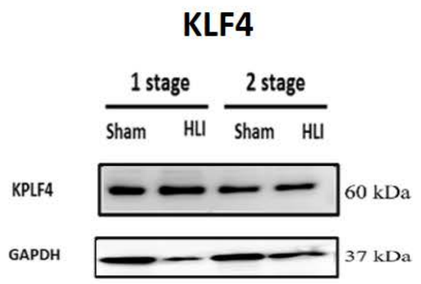

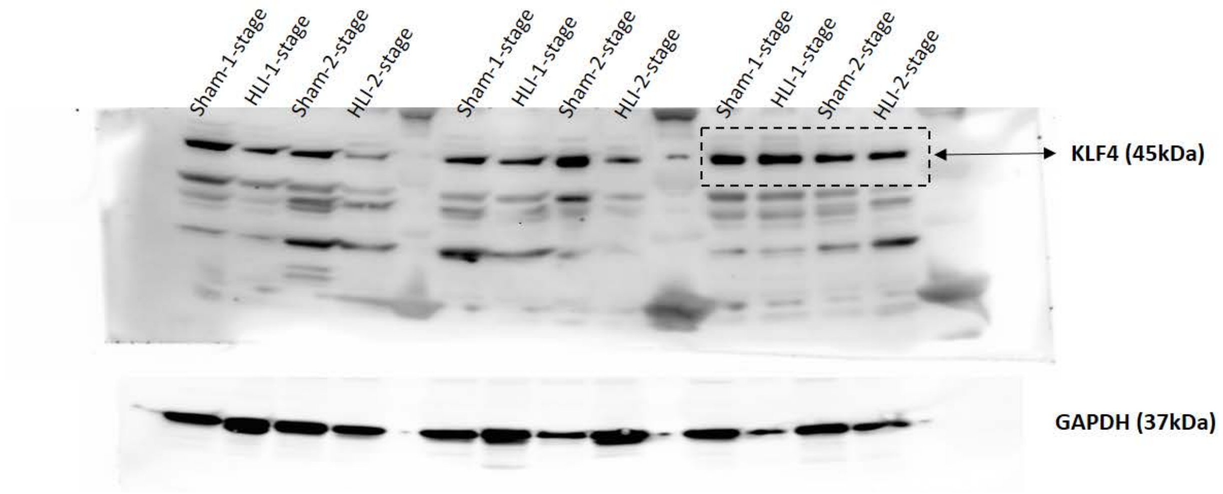

Full un-edited gels of Figure 6 A.

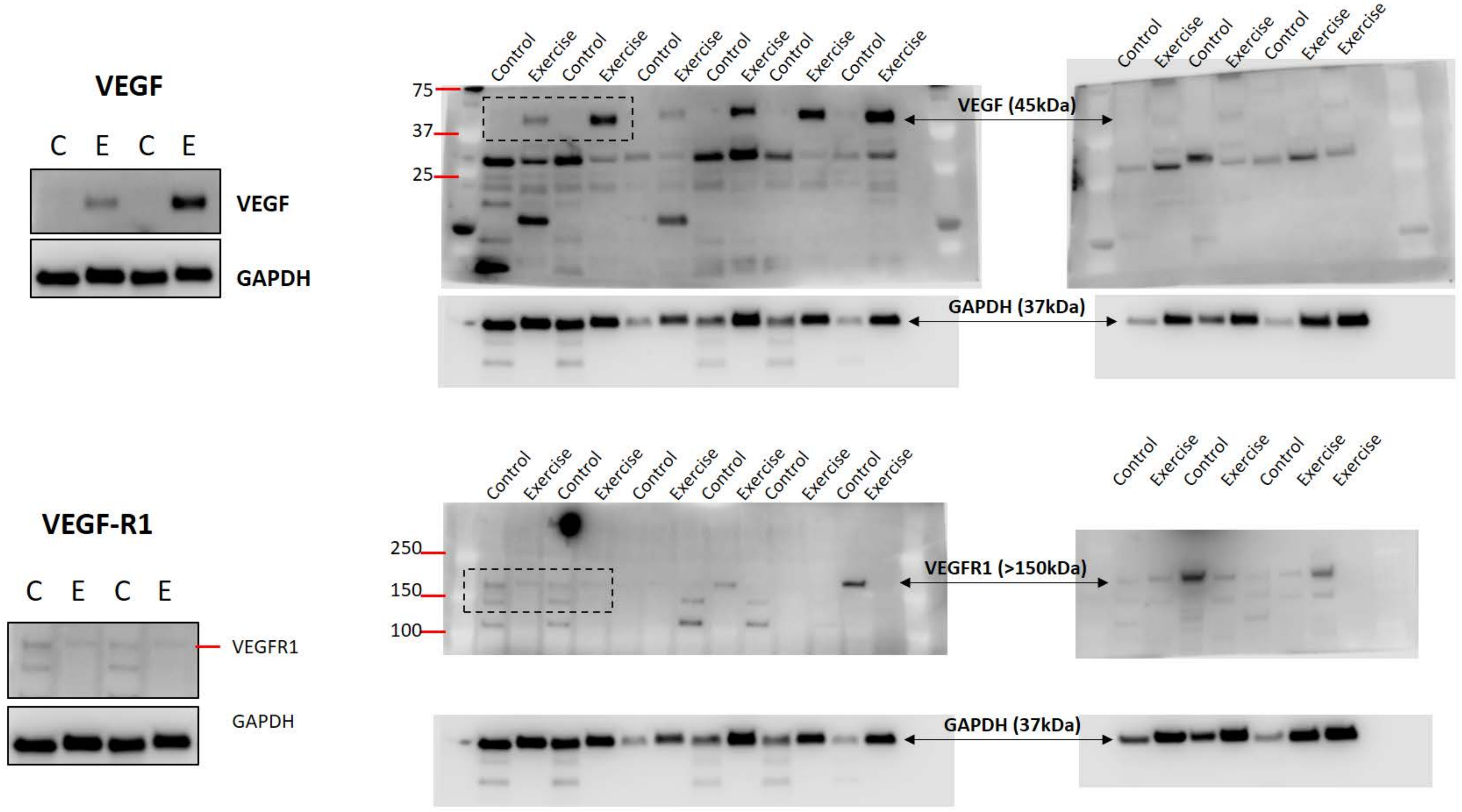

Full un-edited gels of Figure 6 A.

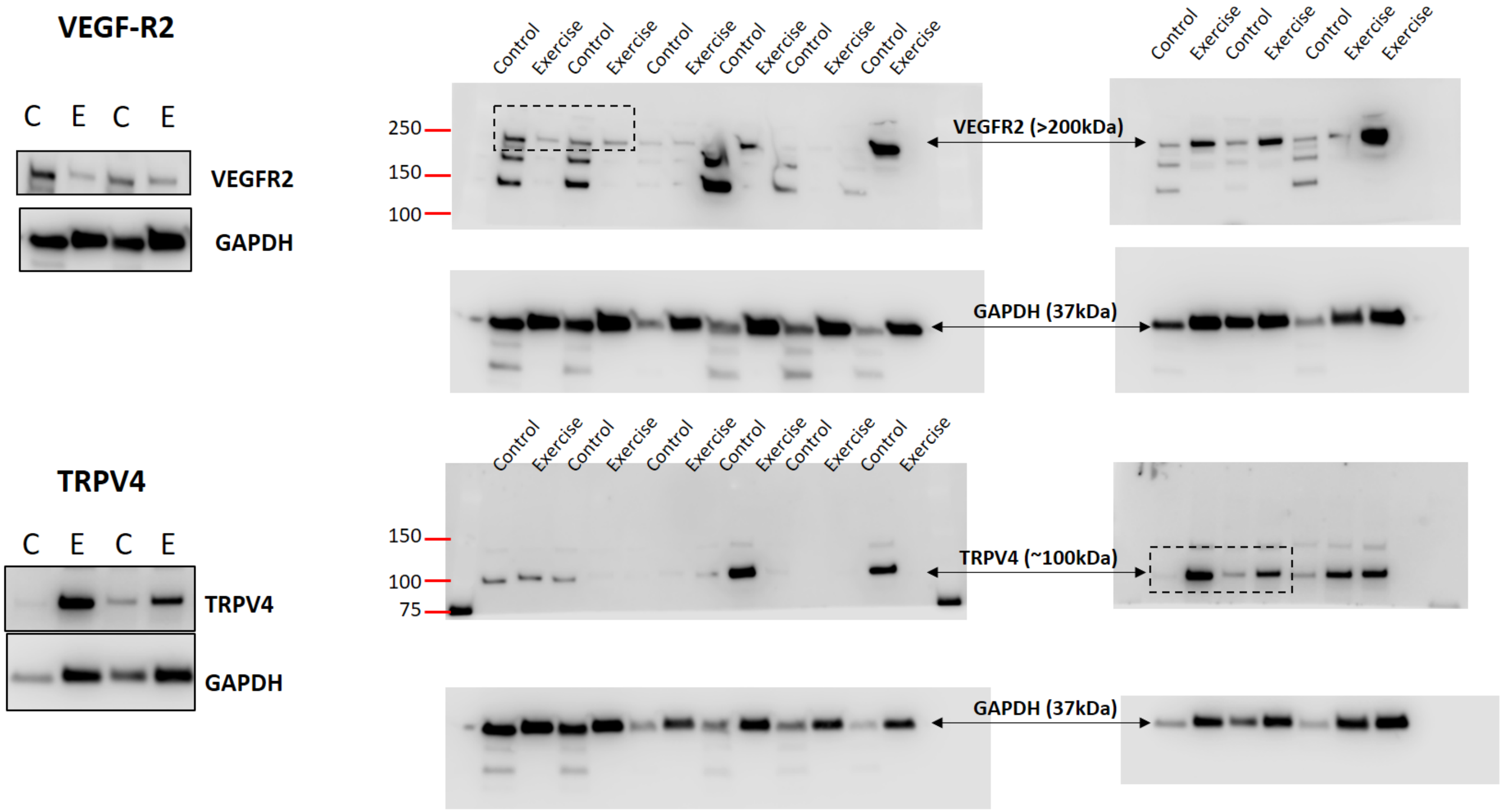

Full un-edited gels of Figure 6 A.

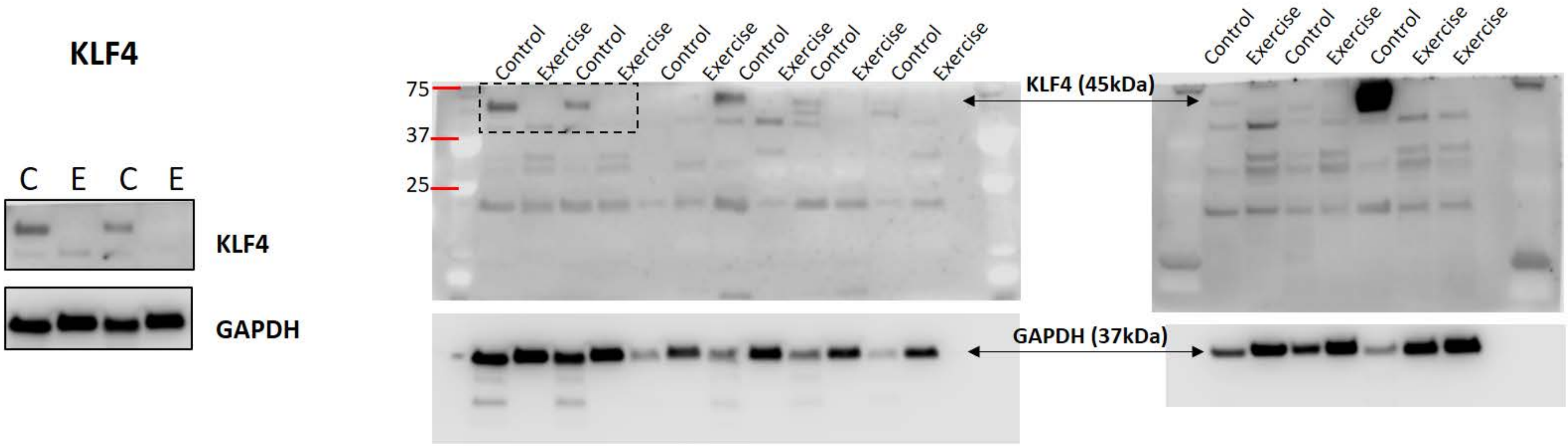

Supplement: Supplementary file 1 — Supplementary Information. [file 41598_2020_60352_MOESM1_ESM.pdf]
